# Diversified Mammalian Visual Adaptations to Bright- or Dim-Light Environments

**DOI:** 10.1093/molbev/msad063

**Published:** 2023-03-17

**Authors:** Yulin Gai, Ran Tian, Fangnan Liu, Yuan Mu, Lei Shan, David M Irwin, Yang Liu, Shixia Xu, Guang Yang

**Affiliations:** Jiangsu Key Laboratory for Biodiversity and Biotechnology, College of Life Sciences, Nanjing Normal University, Nanjing, Jiangsu, China; Jiangsu Key Laboratory for Biodiversity and Biotechnology, College of Life Sciences, Nanjing Normal University, Nanjing, Jiangsu, China; College of Life Sciences, Shaanxi Normal University, Xi’an, Shaanxi, China; College of Animal Science and Veterinary Medicine, Shenyang Agricultural University, Shenyang, Liaoning, China; Jiangsu Key Laboratory for Biodiversity and Biotechnology, College of Life Sciences, Nanjing Normal University, Nanjing, Jiangsu, China; Jiangsu Key Laboratory for Biodiversity and Biotechnology, College of Life Sciences, Nanjing Normal University, Nanjing, Jiangsu, China; Department of Laboratory Medicine and Pathobiology, University of Toronto, Toronto, Ontario, Canada; College of Life Sciences, Shaanxi Normal University, Xi’an, Shaanxi, China; Jiangsu Key Laboratory for Biodiversity and Biotechnology, College of Life Sciences, Nanjing Normal University, Nanjing, Jiangsu, China; Jiangsu Key Laboratory for Biodiversity and Biotechnology, College of Life Sciences, Nanjing Normal University, Nanjing, Jiangsu, China; Southern Marine Science and Engineering Guangdong Laboratory (Guangzhou), Guangzhou, Guangdong, China

**Keywords:** mammals, visual pigment, light environment, molecular evolution, functional assay

## Abstract

Photic niche shifts of mammals are associated with changing visual capabilities, primarily mediated by three visual pigments, two (SWS1 and M/LWS) of them for color vision and rhodopsin (RH1) for dim-light vision. To further elucidate molecular mechanisms of mammalian visual adaptations to different light environments, a systematic study incorporating evolutionary analyses across diverse groups and in vitro assays have been carried out. Here, we collected gene sequences for the three opsins from 220 species covering all major mammalian clades. After screening for cone opsin gene losses, we estimated selective pressures on each of the three genes and compared the levels of selection experienced by species living in bright- and dim-light environments. SWS1 pigment is shown to experience accelerated evolution in species living in bright-light environments as has RH1 in aquatic cetaceans, indicating potential shifts for ecological adaptations. To further elucidate the functional mechanisms for these two pigments, we then carried out site-directed mutagenesis in representative taxa. For SWS1, violet and ultraviolet sensitivities in the pika and mouse are mainly affected by substitutions at the critical sites 86 and 93, which have strong epistatic interaction. For RH1, the phenotypic difference between the sperm whale and bovine sequences is largely contributed by a substitution at site 195, which could be critical for dim-light sensation for deep-diving species. Different evolutionary patterns for the visual pigments have been identified in mammals, which correspond to photic niches, although additional phenotypic assays are still required to fully explain the functional mechanisms.

## Introduction

Vision is an important sensory perception for the survival and evolution of vertebrates (Muller et al. 2018; Baldwin and Ko 2020). The diversification of visual capabilities is associated with the adaptation of multiple taxa to drastically different environments, including extreme niches such as the deep sea (Davies et al. 2012; Hauser and Chang 2017). In vertebrates, vision is initiated through the detection of photons by visual pigments, which are opsins with an individually bound chromophore (Yokoyama 2008). The genome of early vertebrate likely contained five kinds of opsin genes, with four of them expressed in cones that mediate bright-light/color vision, and rhodopsin (RH1), which is expressed in rods and accounts for dim-light vision (Collin et al. 2003; Pisani et al. 2006). However, on the early mammalian lineages, several cone opsin genes were pseudogenized during their adaptation to crepuscular and nocturnal activities (Davies et al. 2012; Borges et al. 2018; Liu et al. 2018, 2019). Thus, many living mammals only possess two cone pigments, that is, either short wavelength-sensitive type 1 (SWS1) and middle/long wavelength-sensitive (M/LWS) pigments in therians or short wavelength-sensitive type 2 (SWS2) and M/LWS pigments in monotremes (Wakefield et al. 2008).

Except for *RH1*, genomes of early therian mammals only contain *SWS1* and *M/LWS* opsin genes (Davies et al. 2012), which is in accordance with their inferred nocturnal lifestyle (Liu et al. 2019). Interestingly, further losses of cone pigment genes (SWS1 and/or M/LWS) are also recorded in diverse mammalian groups that live in dim-light environments, such as nocturnal, fossorial, or deep-diving species (Jacobs 2013; Emerling and Springer 2014, 2015; Springer et al. 2016). Thus, many mammalian species are cone monochromatic, such as some bats that have lost the *SWS1* gene (Zhao, Rossiter et al. 2009; Li et al. 2018; Sadier et al. 2018), and a few other mammalian taxa, like some whales, which have lost both of their cone pigment genes and thus are rod monochromatic (Meredith et al. 2013; Emerling and Springer 2015). On the other hand, duplication of the *M/LWS* gene in Old World monkeys and apes, and also the New World Howler monkey, gave rise to the unique trichromatic color vision found in primates (Hunt et al. 1998).

The ancestral state for SWS1 pigment in mammals is inferred to be ultraviolet (UV)-sensitive (Emerling et al. 2015), with a maximum absorption wavelength (*λ*_max_) of about 360 nm (Shi and Yokoyama 2003; Yokoyama et al. 2016). However, along with the invasion of many taxa into brighter photic environments, the spectral tuning of the SWS1 pigment shifted to be violet/blue-sensitive (> 400 nm) (Emerling et al. 2015). The change of SWS1 pigment spectral tuning between UV and violet sensitivity largely depends on amino acid replacements at 17 critical sites (Yokoyama et al. 2016). In most mammals, positions 86 and 93 are the major critical ones, and normally have epistatic functional interactions with other key sites (Shi et al. 2001; Hauser et al. 2014). For early mammals, the phenotype of the M/LWS pigment has been under debate as to whether it was a red- (∼560 nm) or a green-sensitive (∼530 nm) pigment (Yokoyama and Radlwimmer 1999, 2001). More recent evidence, which included greater species sampling, suggests that it was a yellow-sensitive (∼550 nm) pigment at the origin of mammals (Liu et al. 2018). Subsequently, diversification of M/LWS pigment spectral sensitivities ranging from blue-green- (490 nm) to red-sensitive (∼560 nm) has occurred in different clades that involved changes at more than five critical sites (Yokoyama and Radlwimmer 1999; Liu et al. 2018; Chi et al. 2020).

For dim-light vision, the spectral tuning of rhodopsin has been reported to be conserved across most taxa (with a *λ*_max_ of ∼500 nm) with exceptions mainly observed in some aquatic species due to substitutions at a subset of critical sites (Yokoyama et al. 2008). The spectral sensitivity of rhodopsin from some deep-diving whales and seals was reported to be blue-shifted (< 490 nm), which could be adaptive to the blue light found in the deep sea (Fasick and Robinson 2000; Southall et al. 2002). Apart from spectral tuning, the retinal release rate for rhodopsin is also an important phenotype of pigments for dim-light sensitivity (Chen et al. 2012). Changes in retinal release rates could be adaptive for mammals living in different niches, such as slower release rates reported in nonecholocating fruit bats that might aid in nocturnal activities (Gutierrez, Castiglione et al. 2018). Moreover, the tetrapod ancestor is inferred to have a slower rhodopsin retinal release rate compared with their common ancestor with lungfishes, supporting a nocturnal origin (Liu et al. 2019). On the other hand, faster release rates evolved in deep-diving whales and seals compared with their terrestrial relatives, which might allow them to see rapid changes in light levels as they dive in deep water (Xia et al. 2021; Dungan and Chang 2022).

Although cases have been reported in diverse mammalian clades showing associations between visual pigments and ecology (Zhao, Ru et al. 2009; Dungan et al. 2016; Wu et al. 2017; Gutierrez, Schott et al. 2018; Kries et al. 2018), the molecular mechanisms underlying photic adaptations of mammals are still not fully resolved. In this study, we analyzed the *SWS1*, *M/LWS*, and *RH1* sequences from more than 200 mammalian species, and then compared the selective pressures acting upon these sequences between species that live in bright-light and dim-light environments. Moreover, we expressed opsins in vitro and introduced mutations into the wild-type sequences to yield new insights into the functional mechanisms for visual pigments in mammals.

## Results and Discussion

### Opsin Sequences from Mammals

Coding sequences of 219 *SWS1*, 192 *M/LWS*, and 219 *RH1* were obtained, of which 80, 98, and 86 sequences of each of the three genes, respectively, were annotated in this study. The opsin genes are from a total of 220 species, across all major clades (22 orders) of the class Mammalia. The focal mammalian species were divided into those that live either in a bright-light (83 species) or dim-light (137 species) environment, based on published references (Bennie et al. 2014; Borges et al. 2018) (supplementary table S1, Supplementary Material online). Fossorial, aquatic, and semi-aquatic species were considered to live in dim-light niches (Herbin et al. 1994; Zhao, Ru et al. 2009).

### Pseudogenization of Cone Opsin Genes in Mammals

**Fig. 1.**
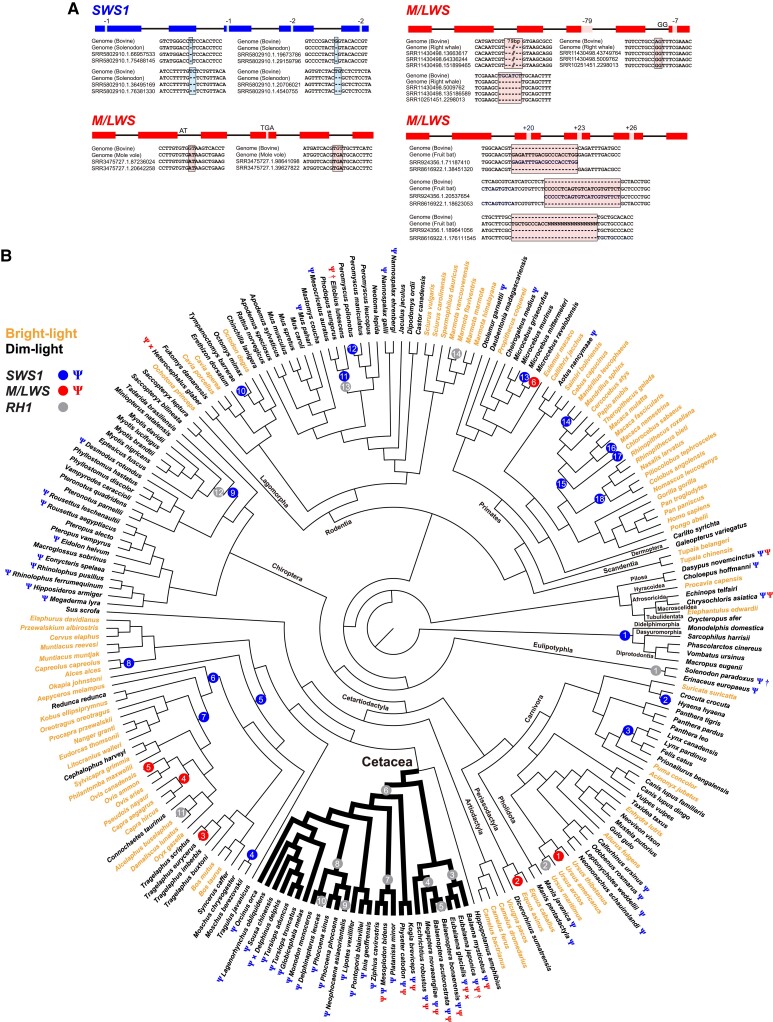
Pseudogenization events in mammalian opsin genes and the selective pressure acting on intact genes. (*A*) The four potential pseudogenes reported here were verified by aligning raw sequence data. *SWS1* and *M/LWS* exons are shown in blue and red, respectively. Bovine opsin sequences were used as references. (*B*) Cone opsin (*SWS1* and *M/LWS*) losses across mammalian species (Ψ), with “†” indicating identified in this study and “×” for no sequence available or poor sequence quality (Meredith et al. 2013; Emerling and Springer 2015). Branches with ω > 1 are indicated by blue (*SWS1*), red (*M/LWS*), and gray (*RH1*) circles. The branch numbers in each circle correspond to that shown in supplementary table S4, Supplementary Material online. Mammalian species living in bright- and dim-light environments are shown in orange and black names, respectively. Branches leading to cetaceans (fully aquatic) are shown in bold.

To verify four newly identified pseudogenes in this study, putative inactivating mutations were confirmed by checking raw sequence reads used to assemble these gene sequences for the corresponding species. The four pseudogenes examined were one *SWS1* from the Hispaniolan solenodon (*Solenodon paradoxus*) and three *M/LWS* genes from the North Pacific right whale (*Eubalaena japonica*), straw-colored fruit bat (*Eidolon helvum*) and Transcaucasian mole vole (*Ellobius lutescens*). Raw sequence reads were retrieved from the Sequence Read Archive (SRA) database by BLAST searching with the putative pseudogene sequences. Of the putative inactivating mutations in these four genes, three could be confirmed, but the raw sequence reads for the *M/LWS* from the straw-colored fruit bat suggest that inactivating mutations in the assembled gene are due to polymorphisms or assembly errors (fig. 1 and supplementary table S2, Supplementary Material online).

In addition to the hedgehog from order Eulipotyphla (Emerling et al. 2015), our analyses indicate that the Hispaniolan solenodon, an echolocating species that shows cases of visual gene loss (Gesemann and Neuhauss 2020; Indrischek et al. 2022), has also lost its *SWS1* gene. For *M/LWS*, the North Pacific right whale, as also reported for other right whales (Meredith et al. 2013; Schweikert et al. 2016), has lost its opsin gene. Species from only a few mammalian groups have no *M/LWS* gene, which include some species from the fully aquatic mammalian clade—whales (Springer et al. 2016), and several subterranean rodents with regressed eyes (Emerling and Springer 2015). The Transcaucasian mole vole is a rodent that lives in a subterranean environment and has small eyes (Herbin et al. 1994) and our analysis revealed that it has also lost its *M/LWS* opsin gene. Thus, in addition to the naked mole rat (*Heterocephalus glaber*) (Emerling and Springer 2015), the Transcaucasian mole vole is another mammalian species that possesses an inactivated *M/LWS* but intact *SWS1* gene. However, to determine whether it is cone (SWS1) or rod monochromatic requires further investigation on other key genes involved in cone phototransduction, as was previously carried out in the naked mole rat (Emerling and Springer 2015).

Together with published inactivated opsin genes, a total of 53 and 12 potential *SWS1* and *M/LWS*, respectively, pseudogene sequences were identified. Details of the inactivating mutation for these genes are listed in supplementary table S3, Supplementary Material online. Relaxed purifying selections were shown for both the *SWS1* and *M/LWS* pseudogenes (*SWS1*: *K* = 0.03, *P* < 0.001; *M/LWS*: *K* = 0.2, *P* < 0.001) when tested with RELAX, using all functional genes as a background. This conclusion was further supported with a small data set, which minimizes the effect of the large number of background branches (see Materials and Methods) (*SWS1*: *K* = 0.01, *P* < 0.001; *M/LWS*: *K* = 0.37, *P* < 0.001). Relaxation of selection pressure on the pseudogene lineages was also examined for the small data set using CODEML (PAML 4) (Yang 2007). The one-ratio model assumes the same levels of selective pressure (*ω*) acts across all taxa and showed that both cone opsin genes are experiencing purifying selection in mammals, a conclusion that is also supported by evidence of sequence conservation (Upton et al. 2021). However, the two-ratio model, which sets independent *ω* values for the pseudogene and the functional gene lineages, shows that the pseudogenes found in dim-light dwelling species have significantly higher ω values compared with the intact genes (table 1). This strengthens the conclusion for relaxed purifying selection acting on cone opsin genes during their adaptation to low-light environments.

**Table 1.** Relaxed Purifying Selection on Pseudogenized *SWS1* and *M/LWS* Genes.

Gene	Model	*ω* value	*ℓ*	*P* value
*SWS1*	One-ratio	*ω* = 0.25	−19,929.26	
	Two-ratio: inactivated *SWS1* genes (ω_1_) and the others (ω_0_)	*ω*_0_ = 0.19 *ω*_1_ = 0.48	−19,857.0	<0.001
*M/LWS*	One-ratio	*ω* = 0.11	−13,213.83	
	Two-ratio: inactivated *M/LWS* genes (ω_1_) and the others (ω_0_)	*ω*_0_ = 0.09 *ω*_1_ = 0.32	−13,169.5	<0.001

### Diversified Selection on Mammalian Opsin Genes

To test whether different levels of selective pressures were exerted on the opsin genes from species living in bright- and dim-light environments, ω values were estimated using CODEML for only functional *SWS1*, *M/LWS*, and *RH1* genes, that is, without the identified pseudogenized *SWS1* and *M/LWS* genes (supplementary table S3, Supplementary Material online). Strong purifying selection was predicted using the one-ratio model, with *ω* values of 0.15 (*SWS1*), 0.1 (*M/LWS*), and 0.04 (*RH1*). However, a likelihood ratio test shows that the free-ratio model fits the data better than the one-ratio model for all three genes (*P* < 0.01), suggesting that heterogeneous selective pressure acts on the different mammalian lineages. As previously reported (Zhao, Ru et al. 2009; Invergo et al. 2013; Fernandez-Sampedro et al. 2016; Wu et al. 2017; Kries et al. 2018), positive selection potentially occurs on some branches. For each of the opsin genes, signals for positive selection identified in species inhabiting both bright-light and dim-light niches, suggesting diversified adaptation to different light environments (fig. 1 and supplementary table S4, Supplementary Material online). Interestingly, evidence for positive selection for all three opsin genes was also reported in another clade of vertebrates, the snakes, the ancestor of which shares the same opsin repertoire as early therian mammals and who have also evolved through a period of dim-light adaptation (Simoes et al. 2016).

**Fig. 2.**
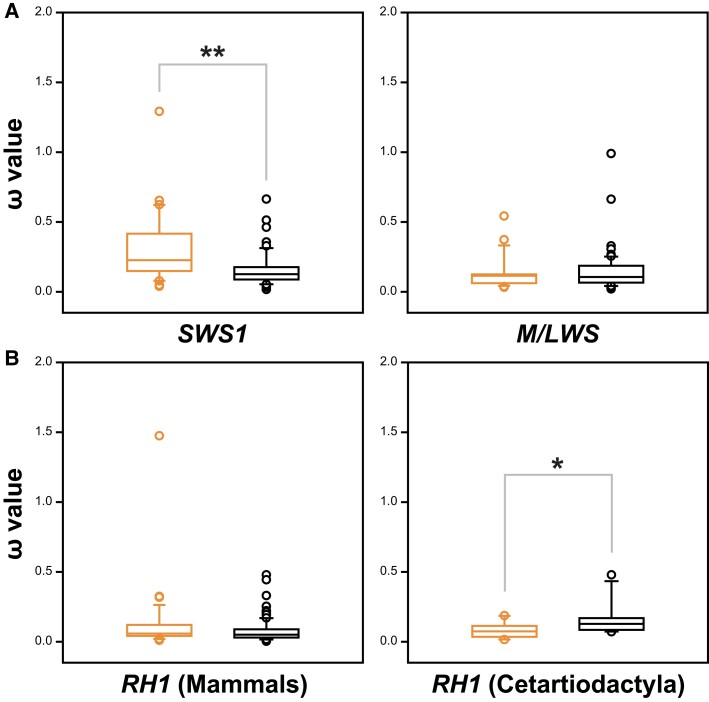
Comparisons of the selective pressures experienced between bright- and dim-light species, with ω values calculated by *d*_N_ or *d*_S_ equaling zero omitted. (*A*) Differences in selective pressures for cone opsin genes (orange for bright-light species and black for dim-light ones), with “*” indicating *P* < 0.01. (*B*) Selection pressures experienced by *RH1* for species living in either bright- or dim-light environments, together with a comparison between *RH1* from whales (black) versus bright-light species (orange) from order Cetartiodactyla (“*”, *P* < 0.05).

To examine the relationships between opsin genes and photic niches, we compared the selective pressures (under the free-ratio model) experienced by these genes in groups of species living in different ecologies. For *SWS1*, significantly higher *ω* values (*P* < 0.001, Mann–Whitney rank-sum test) are observed in species living in bright-light environments (fig. 2), suggesting a functional differentiation compared with *SWS1* genes from species living in dim-light environments. Since early mammals evolved from an ancestor that was active during the crepuscular and nocturnal periods (Gerkema et al. 2013; Liu et al. 2018), accelerated evolution of SWS1 pigments could be adaptive for these species as they started to face brighter niches. It is hypothesized that species that moved to bright-light environments shifted their spectral tuning from UV-sensitive to violet-sensitive (Emerling et al. 2015). Another difference in selection was observed in the *RH1* genes of Cetacea, specifically, significantly higher selective pressures (*P* = 0.019, Mann–Whitney rank-sum test) were found in whale *RH1* compared with genes from their Artiodactyl relatives (fig. 2). This suggests an adaptive functional shift of rhodopsin for the fully aquatic mammalian group, in addition to the previously reported positively selected sites (Meredith et al. 2013; Dungan et al. 2016).

### Spectral Tuning of Pika and Mouse SWS1

**Fig. 3.**
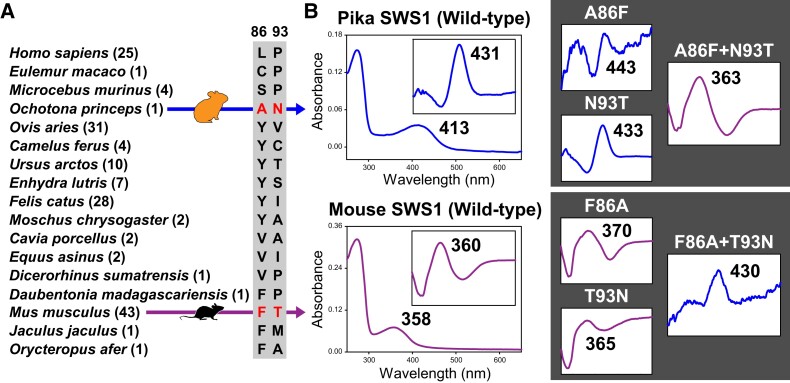
Functional analysis of SWS1 pigments from species in Glires. (*A*) Alignment of the critical sites 86 and 93 in mammalian SWS1 opsin sequences. The combination of the two critical sites in pika (bright light) and mouse (dim-light) is highlighted in red. The number of species for each of the critical site combinations is shown in parentheses. (*B*) In vitro assays for wild-type SWS1 pigments from pika and mouse and for mutants (dark background), with values of *λ*_max_ (nm) shown. Blue or purple lines indicate either violet- or UV-sensitive. Difference spectra are shown for the mutants and also as in the inset plot for wild-type pigments.

By aligning *SWS1* coding sequences from representative mammals living in either bright-light or dim-light niches, multiple substitutions were found at two key sites, positions 86 and 93 (fig. 3). The violet and UV sensitivity shifts of SWS1 could be roughly predicted based on experimentally verified critical sites, especially 86 and 93 in mammals (Shi et al. 2001; Carvalho et al. 2012; Hauser et al. 2014; Yokoyama et al. 2016). The SWS1 pigments in species from order Lagomorpha show a unique residue combination at the two critical sites (A86 and N93). Although electroretinogram measurements (425 nm) have been conducted for the rabbit (Nuboer et al. 1983), the functional impact of the combination of substitutions at these two sites (A86 and N93) and their effect on spectral tuning of SWS1 pigment from American pika (*Ochotona princeps*) (Emerling et al. 2015) is not known. To further evaluate the functional mechanism of pika (bright-light) SWS1, we analyzed wild-type (F86 and T93) and mutant SWS1 pigment from its glire relative, the mouse (who lives in a dim-light environment and has a UV-sensitive SWS1) (Shi et al. 2001; Bennie et al. 2014).

We used site-directed mutagenesis to introduce the amino acid replacements at sites 86 and 93 in both the mouse and pika SWS1 sequences. When a single mutant was introduced into the mouse SWS1 pigment, neither could shift *λ*_max_ from UV-sensitive to violet-sensitive. However, when both pika wild-type residues A86 and N93 in SWS1 were introduced into the mouse opsin, a 70-nm shift to a longer wavelength (*λ*_max_ = 430 nm) was observed, which recovers the spectral tuning of the pika SWS1 pigment. For the reverse mutations, neither single-mutant (A86F or N93T) in the pika *SWS1* pigment showed a UV-sensitive shift. In contrast, the double mutant, of introducing both A86F and N93T, exhibited a large *λ*_max_-shift (−68 nm) to 363 nm (fig. 3). Compared with published mutagenesis results on positions 86 and 93 (Shi et al. 2001; Carvalho et al. 2012; Yokoyama et al. 2016), the reciprocal UV- and violet-sensitive shift seen between pika and mouse SWS1 pigments adds further evidence that A86 and N93 have significant epistatic functional impact, since neither single-mutant at sites 86 or 93 cause a shift between violet and UV sensitivity.

### Shifts of Rhodopsin Retinal Release Rate in Cetaceans

**Fig. 4.**
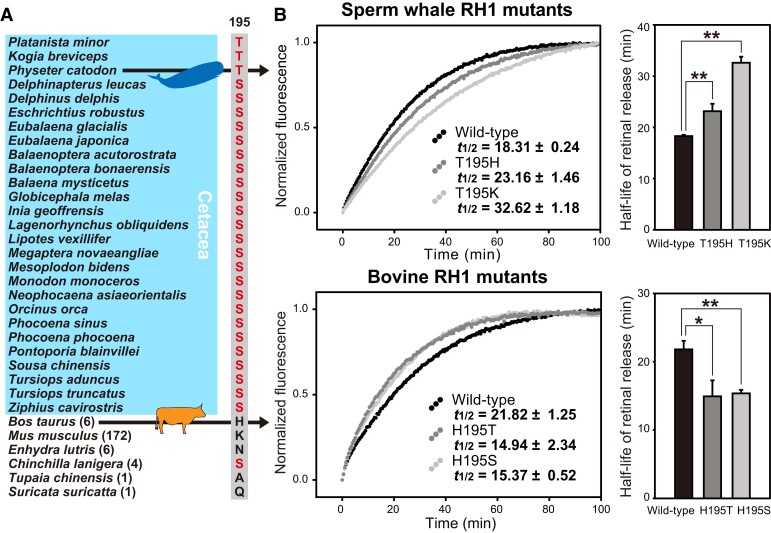
Phenotypic analysis of RH1 pigments from species belonging to Cetartiodactyla. (*A*) Sequence alignment of site 195 in mammalian rhodopsin, with residues that appear in whale rhodopsins (blue background) shown in red. The numbers in parentheses indicate the number of species that share the same residue at this site in RH1. (*B*) Functional impact of mutations at position 195 in sperm whale and bovine rhodopsins, with *t*_1/2_ values (min) calculated. Significant difference between the wild-type and mutant pigments is indicated by “*” for *P* < 0.05 or “*” for *P* < 0.01. The phenotype of bovine wild-type rhodopsin is cited from published data (Liu et al. 2019).

For rhodopsin, we found that the cetacean species, which show significantly higher ω values than its sister group (fig. 2), have different residues at site 195, that is, either threonine (T) or serine (S), compared with other species from Cetartiodactyla (H, K, or N) (fig. 4). Mutagenesis experiments were carried out on the sperm whale *RH1* and the gene in bovine to compare the molecular activity of rhodopsin in these two species that live in dim-light (deep sea) or bright-light environments, respectively. We first introduced residues shown in cetaceans at 195 (T or S) into the bovine *RH1* (H), and found that both mutants (H195T: *λ*_max_ = 500 nm; H195S: *λ*_max_ = 499 nm) show spectral tuning that is similar to wild-type bovine rhodopsin (Liu et al. 2019). We then modified the rhodopsin of the deep-diving sperm whale by changing residue 195 to the two residues (H and K) existing in terrestrial species, and found no difference in spectral tuning between the wild-type sperm whale pigment (T195: *λ*_max_ = 482 nm) or the two mutants (T195H: *λ*_max_ = 482 nm; T195K: *λ*_max_ = 482 nm; supplementary fig. S1, Supplementary Material online). Next, we measured the retinal release rates for the wild-type and mutant pigments. Compared with the published half-life of retinal release in bovine (*t*_1/2_ = 21.82 ± 1.25 min) (Liu et al. 2019), the two rhodopsin mutants have significantly faster retinal release rates (H195T: 14.94 ± 2.34 min, *P* = 0.011; H195S: 15.37 ± 0.52 min, *P* = 0.001, two-tailed *t*-test). No significant difference was observed between the two cetacean substitutions, H195T and H195S, at this site (*P* = 0.774). In contrast, the reverse mutant RH1 pigments from sperm whale lead to significantly slower retinal release rates (T195H: 23.16 ± 1.46 min, *P* = 0.005; T195K: 32.62 ± 1.18 min, *P* < 0.001), compared with wild-type sperm whale rhodopsin (18.31 ± 0.24 min, fig. 4).

Whales, especially deep-diving species, possess rhodopsin pigments with significantly faster retinal release rate compared with their common ancestor with hippos (Xia et al. 2021). Among whales, the sperm whale is an extreme deep-diving species (Collum and Fritts 1985), and has lost both cone pigment genes, which means their vision is completely dependent on the dim-light sensing rhodopsin (Meredith et al. 2013). The faster rhodopsin retinal release rate seen in the sperm whale, compared with the bovine rhodopsin (*P* = 0.009, two-tailed *t*-test), further supports the hypothesis that this phenotypic change is associated with deep-diving and could be adaptive to the rapidly changing light levels as whales dive from surface waters to the deep sea (Dungan and Chang 2017, 2022; Xia et al. 2021). A previous study reported that site 195, together with sites 194 and 292, shows epistatic effects on spectral tuning (Yokoyama et al. 2008). Thus, our results reveal that site 195 in rhodopsin is important for kinetics; however, epistatic interactions with other critical sites, such as sites 83 and 292, are still not clear. In addition to sites 83 and 292, the amino acid substitution at site 195 may also have occurred in the common ancestor of cetaceans (Meredith et al. 2013; Dungan 2018; Xia et al. 2021; Dungan and Chang 2022), suggesting that amino acid substitutions at this site were also crucial during the origin of cetaceans.

## Conclusions

Pseudogenization of the *SWS1* and *M/LWS* gene is generally found in species living in dim-light environments, such as deep-sea diving, nocturnal, or fossorial lifestyles. For functional opsin genes, diversified selection pressures on *SWS1* and *RH1* were found between mammals living in different photic environments, indicating potential visual adaptations. Through our in vitro phenotypic verification of both wild-type and mutant visual pigments, we report new epistatic interactions between residues at sites 86 and 93 of SWS1 explains differentiated adaptation to bright- and dim-light environments in Glires. For RH1, our mutagenesis experiments reveal that 195 in whales is also critical for a retinal release rate shift during the evolution of cetaceans (substitutions to T or S), which could be important for allowing quick dim-light adaptation when diving, although additional mutagenesis experiments are necessary to fully elucidate the epistatic interactions between this site and other known critical substitutions.

## Materials and Methods

### Acquisition of Mammalian Opsin Coding Sequences

We first explored opsin gene sequences (*SWS1*, *M/LWS*, and *RH1*) across 220 mammalian species, representing 22 orders. Coding regions of the three genes were obtained either from GenBank (www.ncbi.nlm.nih.gov) or were annotated by us from the mammalian genomes, with the bovine opsin genes used as queries. Coding sequences (219 for *SWS1*, 192 for *M/LWS*, and 219 for *RH1*) of the three opsin genes were aligned using PRANK (www.ebi.ac.uk/goldman-srv/webprank) (Loytynoja and Goldman 2010) with ambiguous positions in the alignments removed using Gblocks (Talavera and Castresana 2007). Accession numbers for the opsin gene sequences and also taxonomic and ecological information for the species are listed in supplementary table S1, Supplementary Material online.

### Analysis of Inactivated Cone Opsin Genes in Mammals

Published *SWS1* and *M/LWS* pseudogenes have also been verified in this study and are listed in supplementary table S2, Supplementary Material online. The putative pseudogenes identified in this study were aligned using MEGA 6 (Tamura et al. 2013), with the bovine sequences (*SWS1* or *M/LWS*) used as references. Substitutions that led to frame-shift mutations or premature stop codons were validated by retrieving raw sequencing data from the SRA database (www.ncbi.nlm.nih.gov/sra), following procedures described in the literature (Jebb and Hiller 2018; Sharma et al. 2020). Accession numbers for the raw sequencing data used to verify the pseudogenes are shown in supplementary table S3, Supplementary Material online.

Selection intensity for the pseudogenized opsin genes was tested using the RELAX program (Wertheim et al. 2015). Only pseudogene branches leading to tip species were applied as foreground in this analysis, with all other branches treated as background. To exclude a potential effect due to the large number of background genes (intact coding sequences), a second data set was generated with a smaller number (53 species, the same number of species that have inactivated *SWS1*) of intact sequences (termed as the small data set). An analysis using the branch model from the CODEML program (Yang 2007) was also performed for the small data set to further assess the selective pressure acting upon the pseudogenes. The ratio (*ω*) of nonsynonymous (*d*_N_) to synonymous (*d*_S_) substitution rates was estimated for genes across all mammalian species. Both one- and two-ratio models were conducted, where the one-ratio model hypothesizes that *ω* value does not vary among lineages, and the two-ratio model assumes different *ω* values exist in the foreground and background branches. A likelihood ratio test was used to compare the results from the two models, and the better fitting model was selected (Yang 1998). The species tree of mammals from the TimeTree database (www.timetree.org) (Kumar et al. 2017) was used for this analysis.

### Comparisons of the Selective Pressure Acting Upon Functional Opsins from Species Living in Bright- and Dim-Light Environments

After excluding pseudogenes, we analyzed the levels of selective pressure acting upon the functional *SWS1* (166), *M/LWS* (180), and *RH1* (219) genes. Using the CODEML program (Yang 2007), we compared the *ω* values calculated under the free-ratio model, which hypothesizes that lineages may have experienced different selection pressures, to the one-ratio model, where all lineages have the same *ω* value. Next, we compared *ω* values for terminal lineages leading to species living in bright-light environments to the *ω* values for terminal lineages leading to species living in dim-light environments. These comparisons were done individually for the *SWS1*, *M/LWS*, or *RH1* genes, with all values for *d*_N_ or *d*_S_ that equaled zero removed.

### Functional Analysis of Representative SWS1 and RH1 Pigments

Coding sequence for the mouse *SWS1* gene was amplified by PCR using eye cDNA (forward primer: 5′ ATG TCA GGA GAG GAT GAC TTT TA 3′; reverse primer: 5′ GTG AGG GCC AAC TTT GCT AG 3′). Coding sequences for pika *SWS1*, bovine *RH1*, and sperm whale *RH1* were synthesized in vitro. Opsin coding sequences were individually ligated into the pcDNA3.1 (Invitrogen) expression vector containing an inframe epitope tag (“TETSQVAPA”) at the C-terminus of the translated opsin. Plasmids containing either *SWS1* or *RH1* coding sequences were individually transfected into HEK293T cells following the Xfect reagent protocol (Clontech). The cells, 48 h after transfection, were then incubated with the chromophore 11-*cis*-retinal at 4 °C to regenerate the visual pigments in vitro. Purification of visual pigments was conducted using the monoclonal antibody Rho 1D4 (The University of British Columbia) according to published experimental procedures (Liu et al. 2018; Xia et al. 2021). Spectral sensitivities (*λ*_max_) of the SWS1 and RH1 pigments were measured by a UV–visible spectrophotometer. For SWS1, the difference spectrum of each pigment was also calculated after either light- or acid-bleaching (Li et al. 2018; Liu et al. 2018). For RH1, retinal release rates were measured at 20 °C by a fluorescence spectrophotometer. The recorded curves were fitted by the formula *y* = *y*_0_ + *a*(1−e^−*bx*^), with all *r*^2^ values being greater than 0.99. The half-live of each retinal release rate (*t*_1/2_ = ln2/*b*) was then calculated (Bickelmann et al. 2015; Xia et al. 2021).

To elucidate the molecular basis for the phenotypic differences of the visual pigments, site-directed mutagenesis was performed using the Fast Site-Directed Mutagenesis kit (Tiangen Biotech). PCR primers for mutagenesis were designed and are listed in supplementary table S5, Supplementary Material online. Both single- and double-mutants based on the pika and mouse *SWS1* (positions 86 and 93), and also the sperm whale and bovine *RH1* (position 195), were constructed and verified by sequencing, and then used for subsequent functional verifications, as described for the experimental procedures for the wild-type visual pigments above. Both *λ*_max_ and *t*_1/2_ data were then compared between the wild-type and mutant pigments.

## Supplementary Material

msad063_Supplementary_Data

## Data Availability

All data analyzed in this study are available in public databases. ***Conflict of interest statement:*** We declare no competing interests.
