# Supplementary material for: Diversified Mammalian Visual Adaptations to Bright- or Dim-Light Environments
Source: Mol Biol Evol. 2023 Mar 17;40(4):msad063. doi: 10.1093/molbev/msad063 (PMC10075062; doi:10.1093/molbev/msad063)
Supplement: msad063_Supplementary_Data [file msad063_supplementary_data.pdf]

Supplementary Materials

Diversified mammalian visual adaptations to bright- or dim-light environments

Yulin Gai, Ran Tian, Fangnan Liu, Yuan Mu, Lei Shan, David M. Irwin, Yang Liu, Shixia Xu and Guang Yang

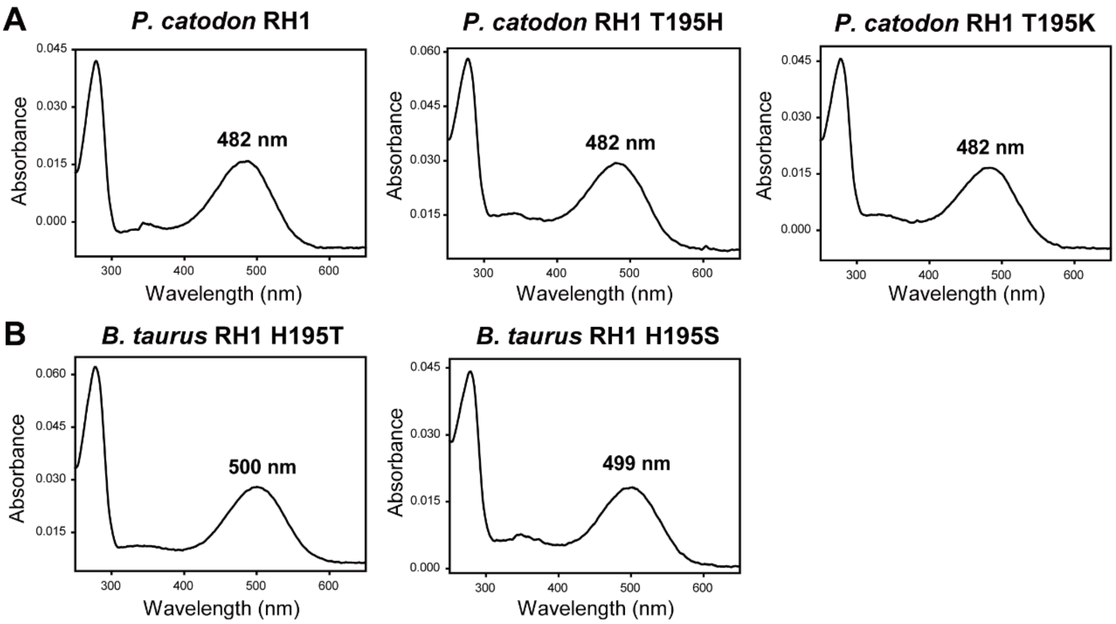

Supplementary fig. S1. Spectral tuning of wild-type and mutant rhodopsins. (A) sperm whale RH1, the λ_{max} of which is close to a published value (Southall, et al. 2002), and its two single-mutants. (B) Bovine RH1 mutants.

Supplementary table S1. Mammalian opsin coding sequences analyzed in this study and the light ecologies of the species

Order	Species	GenBank accession No. *SWS1* (residues at sites 86 and 93)	*M/LWS*	*RH1* (residue at 195)	Genome assembly	References	Light environments Niche	Reference
Dasyuromorphia	*Sarcophilus harrisii*	XM_003771592.3 (F and T)	XM_003774721.2 Genome BLAST	XM_003762449.1 (N)	GCF_902635505.1	(Deeb, et al. 2003; Hunt, et al. 2009; Zhao, Rossiter, et al. 2009; Zhao, Ru, et al. 2009; Emerling, et al. 2015; Ishengoma, et al. 2017; Wu, et al. 2017; Borges, et al. 2018; Gutierrez, Castiglione, et al. 2018; Liu, et al. 2018; Liu, et al. 2019)	Dim-light	(Bennie, et al. 2014)
Didelphimorphia	*Monodelphis domestica*	XM_007504107.2 (F and T)	NM_001145081.1	XM_001366188.2 (K)			Dim-light	(Bennie, et al. 2014)
Diprotodontia	*Macropus eugenii*	AY286017.1 (Y and T)	AY286018.1 Genome BLAST	Genome BLAST (-)	GCA_000004035.1		Dim-light	(Bennie, et al. 2014)
Diprotodontia	*Phascolarctos cinereus*	XM_021002402.1 (F and T)	Genome BLAST	XM_020980962.1 (K)	GCF_002099425.1		Dim-light	(Bennie, et al. 2014)
Diprotodontia	*Vombatus ursinus*	XM_027840210.1 (Y and T)	Genome BLAST	XM_027864063.1 (K)	GCF_900497805.2		Dim-light	(Bennie, et al. 2014)
Cingulata	*Dasypus novemcinctus*	XM_004463203.2	AAGV03294213 AAGV03294214 Genome BLAST	XM_004477246.1 (K)	GCF_000208655.1	(Emerling and Springer 2015; Ishengoma, et al. 2017; Borges, et al. 2018; Gutierrez, Castiglione, et al. 2018; Liu, et al. 2019)	Dim-light	(Bennie, et al. 2014)
Pilosa	*Choloepus hoffmanni*	ABVD02055170 Genome BLAST	ABVD02349172 ABVD02349173 Genome BLAST	ABVD02265667 Genome BLAST (K)	GCA_000164785.2		Dim-light	(Bennie, et al. 2014)
Afrosoricida	*Chrysochloris asiatica*	XM_006861111.1 Genome BLAST	Genome BLAST	XM_006868670.1 (K)	GCF_000296735.1	(Emerling and Springer 2014; Emerling, et al. 2015; Ishengoma, et al. 2017; Wu, et al. 2017; Borges, et al. 2018; Liu, et al. 2018; Liu, et al. 2019)	Dim-light	(Borges, et al. 2018)
Afrosoricida	*Echinops telfairi*	XM_004707803.1 (F and T)	XM_004717562.1	XM_004702378.1 (K)			Dim-light	(Bennie, et al. 2014)
Hyracoidea	*Procavia capensis*	ABRQ02180687 Genome BLAST (Y and T)	Genome BLAST*	KN676591.1 Genome BLAST (K)	GCA_004026925.2		Bright-light	(Bennie, et al. 2014)
Macroscelidea	*Elephantulus edwardii*	XM_006896064.1 (F and T)	XM_006899926.1	XM_006901040.1 (K)			Bright-light	(Bennie, et al. 2014)

Tubulidentata	*Orycteropus afer*	XM_007955413.1 (F and A)	XM_007957152	XM_007956743.1 (K)			Dim-light	(Bennie, et al. 2014)
Cetartiodactyla	*Aepyceros melampus*	Genome BLAST (Y and I)	Genome BLAST	Genome BLAST (K)	GCA_006408695.1	(Jacobs, et al. 1993; Fasick, et al. 1998; Carroll, et al. 2001; Peichl, et al. 2001; Fasick, et al. 2002; Levenson and Dizon 2003; Newman and Robinson 2005; Ahnelt, et al. 2006; Levenson, et al. 2006; Zhao, Rossiter, et al. 2009; Zhao, Ru, et al. 2009; Koito, et al. 2010; McGowen 2011; Meredith, et al. 2013; Zhou, et al. 2013; Emerling and Springer 2014; Emerling, et al. 2015; Emerling and Springer 2015; Dungan, et al. 2016; Springer, et al. 2016; Ishengoma, et al. 2017; Wu, et al. 2017; Borges, et al. 2018; Gutierrez, Schott, et al. 2018; Liu, et al. 2018; Sadier, et al. 2018; Liu, et al. 2019; Simoes, et al. 2019; McGowen, et al. 2020)	Bright-light	(Bennie, et al. 2014)
Cetartiodactyla	*Alcelaphus buselaphus*	Genome BLAST (Y and V)	Genome BLAST	Genome BLAST (-)	GCA_006408545.1		Bright-light	(Bennie, et al. 2014)
Cetartiodactyla	*Alces alces*	Genome BLAST (Y and I)	Genome BLAST	Genome BLAST (K)	GCA_007570765.1		Bright-light	(Bennie, et al. 2014)
Cetartiodactyla	*Bos mutus*	XM_005905247.1 (Y and I)	XM_005909080.1 Genome BLAST	XM_005902834.1 (H)	GCF_000298355.1		Bright-light	(Bennie, et al. 2014)
Cetartiodactyla	*Bos taurus*	NM_174567.1 (Y and I)	NM_174566.1	NM_001014890.2 (H)			Bright-light	(Borges, et al. 2018)
Cetartiodactyla	*Camelus bactrianus*	XM_010947561.1 (Y and C)	XM_010964076.1 Genome BLAST	XM_010953086.1 (K)	GCF_000767855.1		Bright-light	(Borges, et al. 2018)
Cetartiodactyla	*Camelus dromedarius*	XM_010975637.1 (Y and C)	XM_010991460.1	XM_010984900.1 (K)			Bright-light	(Borges, et al. 2018)
Cetartiodactyla	*Camelus ferus*	XM_006184725.1 (Y and C)	XM_006188804.1	XM_006180073.1 (K)			Bright-light	(Bennie, et al. 2014)
Cetartiodactyla	*Capra aegagrus*	Genome BLAST (Y and V)	Genome BLAST	Genome BLAST (K)	GCA_000978405.1		Bright-light	(Bennie, et al. 2014)
Cetartiodactyla	*Capra hircus*	XM_005679445.2 (Y and V)	AH006594 Genome BLAST	XM_018066700.1 (K)	GCF_001704415.1		Bright-light	(Borges, et al. 2018)
Cetartiodactyla	*Capreolus capreolus*	Genome BLAST* (Y and I)	Genome BLAST*	Genome BLAST (K)	GCA_000751575.1		Bright-light	(Bennie, et al. 2014)
Cetartiodactyla	*Cephalophus harveyi*	Genome BLAST (Y and V)	Genome BLAST	Genome BLAST (K)	GCA_006410635.1		Dim-light	(Bennie, et al. 2014)
Cetartiodactyla	*Cervus elaphus*	Genome BLAST (Y and I)	Genome BLAST	Genome BLAST (K)	GCA_002197005.1		Bright-light	(Bennie, et al. 2014)
Cetartiodactyla	*Connochaetes taurinus*	Genome BLAST (Y and V)	Genome BLAST	Genome BLAST (K)	GCA_006408615.1		Dim-light	(Bennie, et al. 2014)
Cetartiodactyla	*Damaliscus lunatus*	Genome BLAST (Y and V)	Genome BLAST	Genome BLAST (K)	GCA_006408505.1		Bright-light	(Bennie, et al. 2014)
Cetartiodactyla	*Elaphurus davidianus*	Genome BLAST (Y and I)	Genome BLAST	Genome BLAST (K)	GCA_002443075.1		Bright-light	(Bennie, et al. 2014)

Cetartiodactyla	*Eudorcas thomsonii*	Genome BLAST (Y and V)	Genome BLAST	Genome BLAST (K)	GCA_006408755.1	Bright-light	(Bennie, et al. 2014)
Cetartiodactyla	*Hippopotamus amphibius*	Genome BLAST* (Y and V)	Genome BLAST	Genome BLAST (K)	GCA_004027065.2	Dim-light	(Bennie, et al. 2014)
Cetartiodactyla	*Kobus ellipsiprymnus*	Genome BLAST (Y and V)	Genome BLAST	Genome BLAST (K)	GCA_006410655.1	Bright-light	(Bennie, et al. 2014)
Cetartiodactyla	*Litocranius walleri*	Genome BLAST (Y and V)	Genome BLAST	Genome BLAST (K)	GCA_006410535.1	Bright-light	(Bennie, et al. 2014)
Cetartiodactyla	*Moschus berezovskii*	Genome BLAST (Y and A)	Genome BLAST	Genome BLAST (K)	GCA_006459085.1	Dim-light	(Bennie, et al. 2014)
Cetartiodactyla	*Moschus chrysogaster*	Genome BLAST (Y and A)	Genome BLAST	Genome BLAST (K)	GCA_006461725.1	Dim-light	(Bennie, et al. 2014)
Cetartiodactyla	*Muntiacus muntjak*	Genome BLAST (Y and I)	Genome BLAST	Genome BLAST (K)	GCA_008782695.1	Bright-light	(Bennie, et al. 2014)
Cetartiodactyla	*Muntiacus reevesi*	Genome BLAST (Y and I)	Genome BLAST	Genome BLAST (K)	GCA_008787405.1	Bright-light	(Bennie, et al. 2014)
Cetartiodactyla	*Nanger granti*	Genome BLAST (Y and V)	Genome BLAST	Genome BLAST (K)	GCA_006408635.1	Bright-light	(Bennie, et al. 2014)
Cetartiodactyla	*Okapia johnstoni*	Genome BLAST* (Y and V)	Genome BLAST*	Genome BLAST* (K)	GCA_001660835.1	Bright-light	(Bennie, et al. 2014)
Cetartiodactyla	*Oreotragus oreotragus*	Genome BLAST (Y and V)	Genome BLAST	Genome BLAST (K)	GCA_006410675.1	Bright-light	(Bennie, et al. 2014)
Cetartiodactyla	*Oryx gazella*	Genome BLAST (Y and V)	Genome BLAST	Genome BLAST (K)	GCA_003945745.1	Bright-light	(Bennie, et al. 2014)
Cetartiodactyla	*Ovis ammon*	Genome BLAST (Y and V)	Genome BLAST	Genome BLAST (K)	GCA_003121645.1	Bright-light	(Bennie, et al. 2014)
Cetartiodactyla	*Ovis aries*	XM_004008047.4 (Y and V)	XM_004022260.3	XM_004018534.4 (K)		Bright-light	(Borges, et al. 2018)
Cetartiodactyla	*Ovis canadensis*	Genome BLAST (Y and V)	Genome BLAST	Genome BLAST (K)	GCA_004026945.1	Bright-light	(Bennie, et al. 2014)
Cetartiodactyla	*Philantomba maxwellii*	Genome BLAST (Y and V)	Genome BLAST	Genome BLAST (K)	GCA_006410695.1	Bright-light	(Bennie, et al. 2014)
Cetartiodactyla	*Procapra przewalskii*	Genome BLAST (Y and V)	Genome BLAST	Genome BLAST (K)	GCA_006410515.1	Bright-light	(Bennie, et al. 2014)

Cetartiodactyla	*Przewalskium albirostris*	Genome BLAST<br>(Y and I)	Genome BLAST	Genome BLAST<br>(K)	GCA_006408465.1	Bright-light	(Bennie, et al. 2014)
Cetartiodactyla	*Pseudois nayaur*	Genome BLAST<br>(Y and V)	Genome BLAST	Genome BLAST<br>(K)	GCA_003182575.1	Bright-light	(Bennie, et al. 2014)
Cetartiodactyla	*Redunca redunca*	Genome BLAST<br>(Y and V)	Genome BLAST	Genome BLAST<br>(K)	GCA_006410935.1	Dim-light	(Bennie, et al. 2014)
Cetartiodactyla	*Sus scrofa*	NM_214090.1<br>(Y and S)	NM_001011506<br>Genome BLAST	NM_214221.1<br>(K)	GCF_000003025.6	Dim-light	(Bennie, et al. 2014)
Cetartiodactyla	*Sylvicapra grimmia*	Genome BLAST<br>(Y and V)	Genome BLAST	Genome BLAST<br>(K)	GCA_006408735.1	Bright-light	(Bennie, et al. 2014)
Cetartiodactyla	*Syncerus caffer*	Genome BLAST<br>(Y and I)	Genome BLAST	Genome BLAST<br>(H)	GCA_902500845.1	Dim-light	(Bennie, et al. 2014)
Cetartiodactyla	*Tragelaphus buxtoni*	Genome BLAST<br>(Y and I)	Genome BLAST	Genome BLAST<br>(H)	GCA_006411685.1	Dim-light	(Bennie, et al. 2014)
Cetartiodactyla	*Tragelaphus eurycerus*	Genome BLAST<br>(Y and I)	Genome BLAST	Genome BLAST<br>(H)	GCA_006410755.1	Dim-light	(Bennie, et al. 2014)
Cetartiodactyla	*Tragelaphus imberbis*	Genome BLAST<br>(Y and I)	Genome BLAST	Genome BLAST<br>(N)	GCA_006410775.1	Dim-light	(Bennie, et al. 2014)
Cetartiodactyla	*Tragelaphus scriptus*	Genome BLAST<br>(Y and I)	Genome BLAST	Genome BLAST<br>(H)	GCA_006410495.1	Dim-light	(Bennie, et al. 2014)
Cetartiodactyla	*Tragulus javanicus*	Genome BLAST<br>(Y and V)	Genome BLAST	Genome BLAST<br>(K)	GCA_004024965.2	Dim-light	(Bennie, et al. 2014)
Cetartiodactyla	*Vicugna pacos*	XM_006202271.1<br>(Y and C)	XM_006218499.1	XM_006206787.1<br>(K)		Bright-light	(Borges, et al. 2018)
Cetartiodactyla	*Balaena mysticetus*	Genome BLAST*	Genome BLAST*	Genome BLAST*<br>(S)	www.bowhead-whale.org	Dim-light	(Borges, et al. 2018)
Cetartiodactyla	*Balaenoptera acutorostrata*	XM_007177192.2	XM_007185533.1<br>Genome BLAST	XM_007192608.1<br>(S)		Dim-light	(Borges, et al. 2018)
Cetartiodactyla	*Balaenoptera bonaerensis*	KX064685.1<br>Genome BLAST	KX118310<br>Genome BLAST	Genome BLAST<br>(S)	GCA_000978805.1	Dim-light	(Borges, et al. 2018)
Cetartiodactyla	*Delphinapterus leucas*	Genome BLAST*	KX118309<br>Genome BLAST	XM_022562745.1<br>(S)	GCF_002288925.2	Dim-light	(Borges, et al. 2018)
Cetartiodactyla	*Delphinus delphis*	-	-	AF055314<br>(S)		Dim-light	(Borges, et al. 2018)

Cetartiodactyla	*Eschrichtius robustus*	Genome BLAST*	KC676824<br>KC676846<br>KC676866<br>KC676887<br>KC676907<br>Genome BLAST	Genome BLAST<br>(S)	GCA_002189225.1	Dim-light	(Borges, et al. 2018)
Cetartiodactyla	*Eubalaena glacialis*	AF545492	-	JQ730751<br>(S)		Dim-light	(Borges, et al. 2018)
Cetartiodactyla	*Eubalaena japonica*	Genome BLAST*	Genome BLAST	Genome BLAST<br>(S)	GCA_004363455.1	Dim-light	(Borges, et al. 2018)
Cetartiodactyla	*Globicephala melas*	XM_030853725.1	XM_030850444.1	XM_030840961.1<br>(S)		Dim-light	(Borges, et al. 2018)
Cetartiodactyla	*Inia geoffrensis*	Genome BLAST	KC676805.1<br>KC676827.1<br>KC676849.1<br>KC676868.1<br>KC676890.1<br>KC676910.1	KC676929.1<br>KC676948.1<br>KC676967.1<br>KC676987.1<br>(S)	GCA_004363515.1	Dim-light	(Borges, et al. 2018)
Cetartiodactyla	*Kogia breviceps*	Genome BLAST	KC676806<br>KC676828<br>KC676850<br>KC676869<br>KC676911<br>Genome BLAST	Genome BLAST<br>(T)	GCA_004363705.1	Dim-light	(Borges, et al. 2018)
Cetartiodactyla	*Lagenorhynchus obliquidens*	Genome BLAST*	XM_027097936.1	XM_027093955.1<br>(S)	GCF_003676395.1	Dim-light	(Borges, et al. 2018)
Cetartiodactyla	*Lipotes vexillifer*	XM_007469685.1	XM_007456663.1	XM_007461564.1<br>(S)		Dim-light	(Borges, et al. 2018)
Cetartiodactyla	*Megaptera novaeangliae*	AY228440<br>Genome BLAST	KC676807<br>KC676829<br>KC676851<br>KC676870<br>KC676891<br>KC676912	Genome BLAST<br>(S)	GCA_004329385.1	Dim-light	(Borges, et al. 2018)

Cetartiodactyla	*Mesoplodon bidens*	Genome BLAST	KC676808<br>KC676830<br>KC676852<br>KC676871<br>KC676892<br>KC676913	AF055316.1<br>Genome BLAST<br>(S)	GCA_004027085.1	Dim-light	(Borges, et al. 2018)
Cetartiodactyla	*Monodon monoceros*	XM_029234347.1	XM_029239868.1	XM_029243393.1<br>(S)		Dim-light	(Borges, et al. 2018)
Cetartiodactyla	*Neophocaena asiaeorientalis*	Genome BLAST	Genome BLAST	Genome BLAST<br>(S)	GCF_003031525.1	Dim-light	(Borges, et al. 2018)
Cetartiodactyla	*Orcinus orca*	Genome BLAST*	XM_004286442.2	XM_004284305.2<br>(S)	GCF_937001465.1	Dim-light	(Borges, et al. 2018)
Cetartiodactyla	*Phocoena sinus*	XM_032643523.1<br>Genome BLAST	Genome BLAST	Genome BLAST<br>(S)	GCA_008692025.1	Dim-light	(Borges, et al. 2018)
Cetartiodactyla	*Phocoena phocoena*	Genome BLAST*	Genome BLAST	KC676933.1<br>KC676952.1<br>KC676971.1<br>KC676989.1<br>(S)	GCA_003071005.1	Dim-light	(Borges, et al. 2018)
Cetartiodactyla	*Physeter catodon*	XM_024132374.1	XM_007113595.1<br>Genome BLAST	XM_007126220.1<br>(T)		Dim-light	(Borges, et al. 2018)
Cetartiodactyla	*Platanista minor*	Genome BLAST	KC676813.1<br>KC676835.1<br>KC676856.1<br>KC676876.1<br>KC676896.1<br>KC676917.1	KC676936.1<br>KC676955.1<br>KC676974.1<br>KC676992.1<br>Genome BLAST<br>(T)	GCA_004363435.1	Dim-light	(Borges, et al. 2018)
Cetartiodactyla	*Pontoporia blainvillei*	Genome BLAST*	KC676814.1<br>KC676836.1<br>KC676857.1<br>KC676877.1<br>KC676897.1<br>KC676918.1	KC676937.1<br>KC676956.1<br>KC676975.1<br>KC676993.1<br>Genome BLAST<br>(S)	GCA_011754075.1	Dim-light	(Borges, et al. 2018)
Cetartiodactyla	*Sousa chinensis*	Genome BLAST*	Genome BLAST	Genome BLAST	GCA_007760645.1	Dim-light	(Borges, et al. 2018)

				(S)			
Cetartiodactyla	*Tursiops aduncus*	Genome BLAST*	Genome BLAST	Genome BLAST<br>(S)	GCA_003227395.1	Dim-light	(Borges, et al. 2018)
Cetartiodactyla	*Tursiops truncatus*	XM_004311115.2	XM_019933644.1	NM_001280659.1<br>(S)		Dim-light	(Borges, et al. 2018)
Cetartiodactyla	*Ziphius cavirostris*	Genome BLAST*	KC676816.1<br>KC676838.1<br>KC676859.1<br>KC676879.1<br>KC676899.1<br>KC676920.1<br>Genome BLAST	KC676938.1<br>KC676957.1<br>KC676977.1<br>KC676995.1<br>Genome BLAST<br>(S)	GCA_004364475.1	Dim-light	(Borges, et al. 2018)
Carnivora	*Acinonyx jubatus*	XM_015071080.2<br>(Y and I)	Genome BLAST	XM_015074629.2<br>(K)	GCF_003709585.1	Bright-light	(Bennie, et al. 2014)
Carnivora	*Ailurus fulgens*	Genome BLAST<br>(Y and S)	Genome BLAST	LNAC01000416.1<br>Genome BLAST<br>(K)	GCA_002007465.1	Bright-light	(Bennie, et al. 2014)
Carnivora	*Callorhinus ursinus*	Genome BLAST	-	XM_025885151.1<br>(K)	GCF_003265705.1	Dim-light	(Borges, et al. 2018)
Carnivora	*Canis lupus dingo*	XM_025471586.1<br>(Y and T)	Genome BLAST	XM_025449310.1<br>(K)	GCA_003254725.2	Dim-light	(Bennie, et al. 2014)
Carnivora	*Canis lupus familiaris*	XM_539386.5<br>(Y and T)	NM_001197072.1	XM_005632035.3<br>(K)		Dim-light	(Bennie, et al. 2014)
Carnivora	*Crocuta crocuta*	Genome BLAST<br>(Y and I)	Genome BLAST	Genome BLAST<br>(K)	GCA_008692635.1	Dim-light	(Bennie, et al. 2014)
Carnivora	*Enhydra lutris*	Genome BLAST<br>(Y and S)	Genome BLAST	Genome BLAST<br>(N)	GCF_002288905.1	Bright-light	(Bennie, et al. 2014)
Carnivora	*Felis catus*	XM_003983019.5<br>(Y and I)	XM_019823330.1	NM_001009242.1<br>(K)		Dim-light	(Borges, et al. 2018)
Carnivora	*Gulo gulo*	Genome BLAST<br>(Y and S)	Genome BLAST	Genome BLAST<br>(N)	GCA_900006375.1	Dim-light	(Bennie, et al. 2014)
Carnivora	*Hyaena hyaena*	Genome BLAST<br>(Y and I)	Genome BLAST	Genome BLAST<br>(K)	GCA_003009895.1	Dim-light	(Bennie, et al. 2014)
Carnivora	*Leptonychotes weddellii*	XM_006745503.1	XM_006739353.1	XM_006740235.1		Dim-light	(Borges, et al. 2018)

				(K)			
Carnivora	*Lynx canadensis*	XM_030295593.1 (Y and I)	Genome BLAST	Genome BLAST (K)	GCF_007474595.1	Dim-light	(Bennie, et al. 2014)
Carnivora	*Lynx pardinus*	Genome BLAST (Y and I)	Genome BLAST	Genome BLAST (K)	GCA_900661375.1	Dim-light	(Bennie, et al. 2014)
Carnivora	*Mustela putorius*	XM_004741814.2 (Y and S)	XM_004780711.1 Genome BLAST	XM_004738577.1 (N)	GCF_000215625.1	Dim-light	(Bennie, et al. 2014)
Carnivora	*Neomonachus schauinslandi*	XM_021699368.1 Genome BLAST	XM_021682937	XM_021695166.1 (K)	GCA_002201575.1	Dim-light	(Borges, et al. 2018)
Carnivora	*Neovison vison*	Genome BLAST (Y and S)	Genome BLAST	Genome BLAST (N)	GCA_900108605.1	Dim-light	(Bennie, et al. 2014)
Carnivora	*Odobenus rosmarus*	XM_004398023.2	XM_004409188.1	XM_004395657.1 (K)		Dim-light	(Borges, et al. 2018)
Carnivora	*Panthera leo*	Genome BLAST (Y and I)	Genome BLAST	Genome BLAST (K)	GCA_008795835.1	Dim-light	(Bennie, et al. 2014)
Carnivora	*Panthera pardus*	XM_019452672.1 (Y and I)	XM_019437761.1	XM_019433593.1 (K)		Dim-light	(Bennie, et al. 2014)
Carnivora	*Panthera tigris*	XM_007078061.2 (Y and I)	Genome BLAST	XM_007075434.1 (K)	GCA_000464555.1	Dim-light	(Bennie, et al. 2014)
Carnivora	*Prionailurus bengalensis*	Genome BLAST (Y and I)	Genome BLAST	Genome BLAST (K)	GCA_005406085.1	Dim-light	(Bennie, et al. 2014)
Carnivora	*Puma concolor*	XM_025929600.1 (Y and I)	Genome BLAST	XM_025920840.1 (K)	GCF_003327715.1	Bright-light	(Bennie, et al. 2014)
Carnivora	*Suricata suricatta*	XM_029955527.1 (Y and I)	Genome BLAST	XM_029917599.1 (Q)	GCF_006229205.1	Bright-light	(Bennie, et al. 2014)
Carnivora	*Taxidea taxus*	Genome BLAST (Y and S)	Genome BLAST	Genome BLAST (K)	GCA_003697995.1	Dim-light	(Bennie, et al. 2014)
Carnivora	*Ursus americanus*	Genome BLAST (Y and T)	Genome BLAST	Genome BLAST (K)	GCA_003344425.1	Bright-light	(Bennie, et al. 2014)
Carnivora	*Ursus arctos*	XM_026510659.1 (Y and T)	Genome BLAST	XM_026501415.1 (K)	GCF_003584765.1	Bright-light	(Bennie, et al. 2014)
Carnivora	*Ursus maritimus*	XM_008705849.1 (Y and T)	XM_008709226 Genome BLAST	XM_008697847.1 (K)		Bright-light	(Bennie, et al. 2014)
Carnivora	*Ursus thibetanus*	Genome BLAST	Genome BLAST	Genome BLAST	GCA_009660055.1	Bright-light	(Bennie, et al. 2014)

		(Y and T)		(K)			
Carnivora	*Vulpes vulpes*	XM_026010889.1<br>(Y and T)	Genome BLAST	XM_026016915.1<br>(K)	GCF_003160815.1	Dim-light	(Bennie, et al. 2014)
Chiroptera	*Desmodus rotundus*	Genome BLAST	MK209520.1<br>Genome BLAST	XM_024556478.1<br>(K)	GCF_002940915.1	Dim-light	(Bennie, et al. 2014)
Chiroptera	*Eidolon helvum*	Genome BLAST	Genome BLAST	Genome BLAST*<br>(K)	GCA_000465285.1	Dim-light	(Bennie, et al. 2014)
Chiroptera	*Eonycteris spelaea*	EU912375.1	-	GQ290305<br>(K)		Dim-light	(Bennie, et al. 2014)
Chiroptera	*Eptesicus fuscus*	XM_008154038.2<br>(F and T)	XM_008159259<br>Genome BLAST	XM_008152292.2<br>(K)	GCF_000308155.1	Dim-light	(Bennie, et al. 2014)
Chiroptera	*Hipposideros armiger*	Genome BLAST	XM_019664260<br>Genome BLAST	NW_017731446.1<br>(K)	GCF_001890085.1	Dim-light	(Bennie, et al. 2014)
Chiroptera	*Macroglossus sobrinus*	Genome BLAST<br>(F and T)	Genome BLAST	Genome BLAST<br>(K)	GCA_004027375.1	Dim-light	(Bennie, et al. 2014)
Chiroptera	*Megaderma lyra*	Genome BLAST	Genome BLAST	KI055942.1<br>Genome BLAST<br>(K)	GCA_004026885.1	Dim-light	(Bennie, et al. 2014)
Chiroptera	*Miniopterus natalensis*	XM_016213323.1<br>(F and T)	XM_016215131.1	XM_016209965.1<br>(K)		Dim-light	(Bennie, et al. 2014)
Chiroptera	*Myotis brandtii*	XM_005868920.2<br>(F and T)	XM_005886120.1<br>Genome BLAST	XM_005870029.2<br>(K)		Dim-light	(Bennie, et al. 2014)
Chiroptera	*Myotis davidii*	XM_006771393.2<br>(F and T)	XM_006766920.2	XM_006758300.2<br>(K)		Dim-light	(Bennie, et al. 2014)
Chiroptera	*Myotis lucifugus*	XM_006088379.2<br>(F and T)	XM_006087664.2	XM_006083811.2<br>(K)		Dim-light	(Bennie, et al. 2014)
Chiroptera	*Myotis nigricans*	MK209554.1<br>(F and T)	MK209508.1	MK209462.1<br>(K)		Dim-light	(Bennie, et al. 2014)
Chiroptera	*Phyllostomus discolor*	XM_028525558.1<br>(F and T)	Genome BLAST	XM_028518094.1<br>(K)	GCF_004126475.1	Dim-light	(Bennie, et al. 2014)
Chiroptera	*Phyllostomus hastatus*	MK209566.1<br>(F and T)	MK209523.1	MK209477.1<br>(K)		Dim-light	(Bennie, et al. 2014)
Chiroptera	*Pteronotus parnellii*	Genome BLAST<br>(F and T)	Genome BLAST	KE878654.1<br>Genome BLAST	GCA_000465405.1	Dim-light	(Bennie, et al. 2014)

				(K)			
Chiroptera	*Pteronotus quadridens*	MG775064.1<br>(F and T)	MK209513.1	MK209467.1<br>(K)		Dim-light	(Bennie, et al. 2014)
Chiroptera	*Pteropus alecto*	XM_006910537.1<br>(F and T)	XM_006915297.1	XM_006917646.1<br>(K)		Dim-light	(Bennie, et al. 2014)
Chiroptera	*Pteropus vampyrus*	XM_011357263.1<br>(F and T)	XM_011375552<br>Genome BLAST	XM_011370659.1<br>(K)		Dim-light	(Bennie, et al. 2014)
Chiroptera	*Rhinolophus ferrumequinum*	Genome BLAST<br>EU912380	Genome BLAST	XM_033132484.1<br>(K)	GCA_004115265.2	Dim-light	(Bennie, et al. 2014)
Chiroptera	*Rhinolophus pusillus*	EU912382.1	-	GQ290315<br>(K)		Dim-light	(Bennie, et al. 2014)
Chiroptera	*Rousettus aegyptiacus*	XM_016148657.1<br>Genome BLAST	XM_016158799.1	XM_016122023.1<br>(K)		Dim-light	(Bennie, et al. 2014)
Chiroptera	*Rousettus leschenaultii*	EU912381.1	EU912350	GQ290317<br>(K)		Dim-light	(Bennie, et al. 2014)
Chiroptera	*Saccopteryx bilineata*	MK209552.1<br>(F and T)	MK209506.1	MK209460.1<br>(K)		Dim-light	(Bennie, et al. 2014)
Chiroptera	*Saccopteryx leptura*	MK209553.1<br>(F and T)	MK209507.1	MK209461.1<br>(K)		Dim-light	(Bennie, et al. 2014)
Chiroptera	*Tadarida brasiliensis*	MK209555.1<br>(F and T)	MK209510.1	MK209464.1<br>(K)		Dim-light	(Bennie, et al. 2014)
Chiroptera	*Vampyrodes caraccioli*	MK209578.1<br>(F and T)	MK209537.1	MK209491.1<br>(K)		Dim-light	(Bennie, et al. 2014)
Pholidota	*Manis javanica*	XM_017652220.1	XM_017640030<br>Genome BLAST	XM_017647349.1<br>(K)		Dim-light	(Bennie, et al. 2014)
Pholidota	*Manis pentadactyla*	JPTV01022983<br>Genome BLAST	JPTV01114000<br>Genome BLAST	KN008348.1<br>Genome BLAST<br>(K)	GCA_000738955.1	Dim-light	(Bennie, et al. 2014)
Eulipotyphla	*Solenodon paradoxus*	Genome BLAST	Genome BLAST	Genome BLAST<br>(K)	GCA_002901085.1	Dim-light	(Bennie, et al. 2014)
Perissodactyla	*Dicerorhinus sumatrensis*	Genome BLAST<br>(V and P)	Genome BLAST	Genome BLAST<br>(K)	GCA_002844835.1	Dim-light	(Bennie, et al. 2014)
Perissodactyla	*Equus caballus*	XM_001502735.5<br>(V and I)	NM_001081845.1	XM_023619934.1<br>(K)		Bright-light	(Borges, et al. 2018)

Perissodactyla	*Equus asinus*	XM_014854379.1 (V and I)	Genome BLAST	XM_014838425.1 (K)	GCF_001305755.1		Bright-light	(Borges, et al. 2018)
Erinaceomorpha	*Erinaceus europaeus*	XM_007526304.1	XM_007534857.1	XM_007517079.1 (K)			Dim-light	(Bennie, et al. 2014)
Dermoptera	*Galeopterus variegatus*	XM_008585390.1 (Y and I)	XM_008578409 Genome BLAST	XM_008574046.1 (K)	GCF_000696425.1	(Wikler and Rakic 1990; Shimmin, et al. 1998; Yokoyama, et al. 1998; Calderone and Jacobs 1999; David-Gray, et al. 2002; Onishi, et al. 2002; Parry, et al. 2004; Tan, et al. 2005; Carvalho, et al. 2006; Levenson, et al. 2007; Zhao, Rossiter, et al. 2009; Carvalho, et al. 2012; Arbogast, et al. 2013; Moritz, et al. 2013; Veilleux, et al. 2013; Emerling and Springer 2014; Fang, et al. 2014; Emerling, et al. 2015; Ishengoma, et al. 2017; Wu, et al. 2017; Borges, et al. 2018; Liu, et al. 2018; Liu, et al. 2019)	Dim-light	(Bennie, et al. 2014)
Scandentia	*Tupaia belangeri*	Genome BLAST (-)	Genome BLAST	-	GCA_000181375.1		Bright-light	(Bennie, et al. 2014)
Scandentia	*Tupaia chinensis*	XM_006146852.1 (Y and V)	XM_006166677 Genome BLAST	XM_006160664.1 (A)	GCF_000334495.1		Bright-light	(Borges, et al. 2018)
Primates	*Aotus nancymaae*	Genome BLAST	Genome BLAST	XM_012472901.2 (K)	GCF_000952055.2		Dim-light	(Bennie, et al. 2014)
Primates	*Callithrix jacchus*	XM_002752031.3 (L and P)	XM_002763410.4	XM_008982215.2 (K)			Bright-light	(Bennie, et al. 2014)
Primates	*Carlito syrichta*	XM_008063590.2 (L and P)	XM_008071543.1	XM_008047820.2 (K)			Dim-light	(Bennie, et al. 2014)
Primates	*Cebus capucinus*	XM_017549511.2 (L and P)	AB193772.1 Genome BLAST	XM_017505088.2 (K)	GCF_001604975.1		Bright-light	(Bennie, et al. 2014)
Primates	*Cercocebus atys*	XM_012087992.1 (L and P)	-	XM_012060295.1 (K)	GCF_000955945.1		Bright-light	(Bennie, et al. 2014)
Primates	*Cheirogaleus medius*	Genome BLAST*	Genome BLAST	Genome BLAST (K)	GCA_008086735.1		Dim-light	(Bennie, et al. 2014)
Primates	*Chlorocebus sabaeus*	XM_007982851.1 (L and P)	-	XM_007985378.1 (K)			Bright-light	(Bennie, et al. 2014)
Primates	*Colobus angolensis*	XM_011939561.1 (L and P)	-	XM_011950692.1 (K)			Bright-light	(Bennie, et al. 2014)
Primates	*Daubentonia madagascariensis*	EF667283 Genome BLAST (F and P)	Genome BLAST	Genome BLAST (K)	GCA_004027145.1		Dim-light	(Bennie, et al. 2014)
Primates	*Eulemur macaco*	Genome BLAST (C and P)	Genome BLAST	Genome BLAST (K)	GCA_001262655.1		Bright-light	(Bennie, et al. 2014)
Primates	*Gorilla gorilla*	XM_004046176.2 (L and P)	-	XM_004036292.2 (K)			Bright-light	(Bennie, et al. 2014)
Primates	*Homo sapiens*	NM_001385125.1	NM_020061.5	NM_000539.3			Bright-light	(Borges, et al. 2018)

		(L and P)		(K)				
Primates	*Macaca fascicularis*	NM_001283204.1 (L and P)	-	NM_001283360.1 (K)			Bright-light	(Bennie, et al. 2014)
Primates	*Macaca mulatta*	XM_001091869.3 (L and P)	-	XM_001094250.2 (K)			Bright-light	(Bennie, et al. 2014)
Primates	*Macaca nemestrina*	XM_024790697.1 (L and P)	-	XM_011734526.1 (K)			Bright-light	(Bennie, et al. 2014)
Primates	*Mandrillus leucophaeus*	XM_011995905.1 (L and P)	-	XM_011974200.1 (K)			Bright-light	(Bennie, et al. 2014)
Primates	*Mandrillus sphinx*	Genome BLAST (L and P)	-	Genome BLAST (K)	GCA_004802615.1		Bright-light	(Bennie, et al. 2014)
Primates	*Microcebus griseorufus*	JX867521 Genome BLAST (S and P)	Genome BLAST	Genome BLAST (K)	GCA_008750995.1		Dim-light	(Bennie, et al. 2014)
Primates	*Microcebus mittermeieri*	Genome BLAST (S and P)	Genome BLAST	Genome BLAST (K)	GCA_008750955.1		Dim-light	(Bennie, et al. 2014)
Primates	*Microcebus murinus*	XM_012789156.1 (S and P)	XM_012757188.1	XM_012785306.2 (K)			Dim-light	(Bennie, et al. 2014)
Primates	*Microcebus ravelobensis*	JX867533 Genome BLAST (S and P)	Genome BLAST	Genome BLAST (K)	GCA_008750975.1		Dim-light	(Bennie, et al. 2014)
Primates	*Nasalis larvatus*	Genome BLAST (L and P)	-	Genome BLAST (K)	GCA_000772465.1		Bright-light	(Bennie, et al. 2014)
Primates	*Nomascus leucogenys*	XM_003261297.2 (L and P)	-	XM_003265030.3 (K)			Bright-light	(Bennie, et al. 2014)
Primates	*Otolemur garnettii*	XM_012805124.1	XM_003802556.1	XM_003796229.3 (K)			Dim-light	(Bennie, et al. 2014)
Primates	*Pan paniscus*	XM_034964951.1 (L and P)	-	XM_003829435.4 (K)			Bright-light	(Bennie, et al. 2014)
Primates	*Pan troglodytes*	NM_001009127.1 (L and P)	-	XM_516740.7 (K)			Bright-light	(Bennie, et al. 2014)
Primates	*Papio anubis*	XM_003896561.4 (L and P)	-	XM_003906878.3 (K)			Bright-light	(Bennie, et al. 2014)

Primates	*Piliocolobus tephrosceles*	XM_023223538.1 (L and P)	-	Genome BLAST (K)	GCF_002776525.2	Bright-light	(Bennie, et al. 2014)
Primates	*Pongo abelii*	XM_002818421.2 (L and P)	-	XM_002813145.4 (K)		Bright-light	(Bennie, et al. 2014)
Primates	*Propithecus coquereli*	XM_012664772.1 (L and P)	XM_012662045.1 Genome BLAST	XM_012655172.1 (K)	GCF_000956105.1	Bright-light	(Bennie, et al. 2014)
Primates	*Rhinopithecus bieti*	XM_017896221.1 (L and P)	-	XM_017885138.1 (K)		Bright-light	(Bennie, et al. 2014)
Primates	*Rhinopithecus roxellana*	XM_010378740.2 (L and P)	-	XM_010381505.2 (K)		Bright-light	(Bennie, et al. 2014)
Primates	*Saimiri boliviensis*	XM_003921010.2 (L and P)	XM_003943902.1	XM_003926159.2 (K)		Bright-light	(Bennie, et al. 2014)
Primates	*Theropithecus gelada*	XM_025379936.1 (L and P)	-	XM_025377237.1 (K)		Bright-light	(Bennie, et al. 2014)
Rodentia	*Apodemus speciosus*	Genome BLAST (F and T)	Genome BLAST	Genome BLAST (K)	GCA_002335545.1	Dim-light	(Bennie, et al. 2014)
Rodentia	*Apodemus sylvaticus*	Genome BLAST (F and T)	Genome BLAST	Genome BLAST (K)	GCA_001305905.1	Dim-light	(Bennie, et al. 2014)
Rodentia	*Castor canadensis*	XM_020160322.1 Genome BLAST (F and T)	XM_020188197 Genome BLAST	NW_017871432.1 (K)	GCF_001984765.1	Dim-light	(Bennie, et al. 2014)
Rodentia	*Cavia aperea*	Genome BLAST (V and A)	Genome BLAST	Genome BLAST (K)	GCA_000688575.1	Bright-light	(Bennie, et al. 2014)
Rodentia	*Cavia porcellus*	NM_001172758.1 (V and A)	AF132042 Genome BLAST	NM_001173085.1 (K)	GCA_000688575.1	Bright-light	(Bennie, et al. 2014)
Rodentia	*Chinchilla lanigera*	XM_005402383.2 (F and T)	XM_005414184.1 Genome BLAST	XM_005387085.2 (S)	GCF_000276665.1	Dim-light	(Bennie, et al. 2014)
Rodentia	*Dipodomys ordii*	XM_013019960.1 (F and T)	XM_013035313.1	XM_013010425.1 (K)		Dim-light	(Bennie, et al. 2014)
Rodentia	*Ellobius lutescens*	Genome BLAST (F and T)	Genome BLAST	Genome BLAST (K)	GCA_001685075.1	Dim-light	(Herbin, et al. 1994)
Rodentia	*Erethizon dorsatum*	Genome BLAST (F and T)	Genome BLAST	Genome BLAST (K)	GCA_006547115.1	Dim-light	(Bennie, et al. 2014)
Rodentia	*Fukomys damarensis*	XM_010633453.2	XM_010628046	XM_010637946.1	GCF_000743615.1	Dim-light	(Borges, et al. 2018)

		(F and T)	Genome BLAST	(K)			
Rodentia	*Heterocephalus glaber*	XM_004856389.1<br>(F and T)	-	XM_004870461.1<br>(K)	GCF_000247695.1	Dim-light	(Borges, et al. 2018)
Rodentia	*Jaculus jaculus*	XM_004661221.2<br>(F and M)	XM_004672101<br>Genome BLAST	XM_004651581.1<br>(K)	GCF_000280705.1	Dim-light	(Bennie, et al. 2014)
Rodentia	*Marmota flaviventris*	XM_027926526.1<br>(Y and V)	Genome BLAST	XM_027931952.1<br>(K)	GCF_003676075.1	Bright-light	(Bennie, et al. 2014)
Rodentia	*Marmota himalayana*	Genome BLAST<br>(Y and V)	Genome BLAST	Genome BLAST<br>(K)	GCA_005280165.1	Bright-light	(Bennie, et al. 2014)
Rodentia	*Marmota marmota*	XM_015488317.1<br>(Y and V)	XM_015506949<br>Genome BLAST	XM_015495128.1<br>(K)	GCF_001458135.1	Bright-light	(Bennie, et al. 2014)
Rodentia	*Marmota vancouverensis*	Genome BLAST<br>(Y and V)	Genome BLAST	Genome BLAST<br>(K)	GCA_005458795.1	Bright-light	(Bennie, et al. 2014)
Rodentia	*Mastomys coucha*	XM_031380794.1<br>(F and T)	Genome BLAST	Genome BLAST<br>(K)	GCF_008632895.1	Dim-light	(Bennie, et al. 2014)
Rodentia	*Mesocricetus auratus*	XM_012805124.1<br>Genome BLAST	XM_005086920<br>Genome BLAST	XM_005066100.4<br>(K)	GCF_000349665.1	Dim-light	(Bennie, et al. 2014)
Rodentia	*Mus caroli*	XM_021164740.1<br>(F and T)	XM_021153781.1	XM_021165399.1<br>(K)		Dim-light	(Bennie, et al. 2014)
Rodentia	*Mus musculus*	NM_007538.3<br>(F and T)	NM_008106.2	NM_145383.2<br>(K)		Dim-light	(Bennie, et al. 2014)
Rodentia	*Mus pahari*	XM_021189852.1	XM_021188393.2	XM_021190968.1<br>(K)		Dim-light	(Bennie, et al. 2014)
Rodentia	*Mus spretus*	Genome BLAST<br>(F and T)	Genome BLAST	Genome BLAST<br>(K)	GCA_001624865.1	Dim-light	(Bennie, et al. 2014)
Rodentia	*Nannospalax ehrenbergi*	AY099455.1	-	AF309568<br>(K)		Dim-light	(Borges, et al. 2018)
Rodentia	*Nannospalax galili*	XM_008846382.1<br>Genome BLAST	XM_008829579.1<br>Genome BLAST	XM_008854920.1<br>(K)	GCF_000622305.1	Dim-light	(Borges, et al. 2018)
Rodentia	*Neotoma lepida*	Genome BLAST<br>(F and T)	Genome BLAST	Genome BLAST<br>(K)	GCA_001675575.1	Dim-light	(Bennie, et al. 2014)
Rodentia	*Octodon degus*	XM_004642726.1<br>(F and T)	XM_004645096<br>Genome BLAST	XM_004645617.3<br>(S)	GCF_000260255.1	Bright-light	(Bennie, et al. 2014)
Rodentia	*Octomys mimax*	Genome BLAST	Genome BLAST	Genome BLAST	GCA_002564305.1	Dim-light	(Bennie, et al. 2014)

		(F and T)		(S)			
Rodentia	*Peromyscus leucopus*	XM_028866221.2<br>(F and T)	Genome BLAST	XM_028864082.2<br>(K)	GCF_004664715.1	Dim-light	(Bennie, et al. 2014)
Rodentia	*Peromyscus maniculatus*	XM_006979426.2<br>(F and T)	XM_006992450.1	XM_006978532.1<br>(K)		Dim-light	(Bennie, et al. 2014)
Rodentia	*Peromyscus polionotus*	Genome BLAST<br>(F and T)	Genome BLAST	Genome BLAST<br>(K)	GCA_003704135.2	Dim-light	(Bennie, et al. 2014)
Rodentia	*Phodopus sungorus*	Genome BLAST<br>(F and T)	Genome BLAST	Genome BLAST<br>(K)	GCA_001707965.1	Dim-light	(Bennie, et al. 2014)
Rodentia	*Rattus norvegicus*	NM_031015.1<br>(F and T)	NM_053548.1	NM_033441.1<br>(K)		Dim-light	(Bennie, et al. 2014)
Rodentia	*Sciurus carolinensis*	DQ302163<br>Genome BLAST<br>(Y and V)	AF132044<br>Genome BLAST	Genome BLAST<br>(K)	GCA_902686445.1	Bright-light	(Bennie, et al. 2014)
Rodentia	*Sciurus vulgaris*	Genome BLAST<br>(Y and V)	Genome BLAST	Genome BLAST<br>(K)	GCA_902686455.1	Bright-light	(Bennie, et al. 2014)
Rodentia	*Spermophilus dauricus*	Genome BLAST<br>(Y and V)	Genome BLAST	Genome BLAST<br>(K)	GCA_002406435.1	Bright-light	(Bennie, et al. 2014)
Rodentia	*Tympanoctomys barrerae*	Genome BLAST<br>(F and T)	Genome BLAST	Genome BLAST<br>(S)	GCA_002564285.1	Dim-light	(Bennie, et al. 2014)
Lagomorpha	*Ochotona princeps*	XM_004592516.1<br>(A and N)	XM_004598859<br>Genome BLAST	XM_004581320.1<br>(K)	GCF_000292845.1	Bright-light	(Bennie, et al. 2014)

* Double-checked genomic annotation in this study.
Underlined sequences were also used in the small dataset.

Supplementary table S2. Raw sequencing data verification of newly identified pseudogenes in this study

Gene	Species	Types of inactivating mutations			SRA accession number	Project No.
		**Frame-shift mutation**	**Premature stop codon**	**Splice site mutation**		
SWS1	*Solenodon paradoxus*	1-bp deletion at position 103			SRR5802910	PRJNA368679
		1-bp deletion at 566				
		2-bp deletion at 780-781				
		2-bp deletion at 1020-1021				
M/LWS	*Ellobius lutescens*		Exon 4: 679-681	Intron 2: GT → AT;	SRR3475727	PRJNA305123
	Eubalaena japonica	79-bp deletion at 666-744		Intron 5: AG → GG	SRR11430498	PRJNA399464
		7-bp deletion at 994-1000			SRR10251451	PRJNA575269
	Eidolon helvum	20-bp insertion at 486-555			SRR924356	PRJNA209406
		23-bp insertion at 714-736			SRR8616922	PRJNA512907
		26-bp insertion at 916-941				

Supplementary table S3. Inactivating mutations in *SWS1* and *M/LWS* genes, together with previously published pseudogenes

Gene	Order	Species	Accession No.	Mutation types	Reference	Newly identified mutation(s)	Data used in this study
SWS1	Cingulata	*D. novemcinctus*	AAGV03126450	Frameshift insertions and deletions<br>Premature stop codons	(Emerling and Springer 2015)	None	XM_004463203.2
	Pilosa	*C. hoffmanni*	ABVD02055170	Frameshift insertions and deletions<br>Premature stop codons	(Emerling and Springer 2015)	None	ABVD02055170<br>Genome BLAST
	Afrosoricida	*C. asiatica*	AMDV01100167	Frameshift deletions	(Emerling and Springer 2014)	None	XM_006861111.1<br>Genome BLAST
	Cetartiodactyla	*B. mysticetus*	KC676998	Frameshift deletion	(Levenson and Dizon 2003)<br>(Meredith, et al. 2013)	None	Genome BLAST
	Cetartiodactyla	*B. acutorostrata*	KC677002	Frameshift deletion	(Levenson and Dizon 2003)<br>(Meredith, et al. 2013)	None	XM_007177192.2
	Cetartiodactyla	*B. bonaerensis*	KX064685.1	Frameshift deletion and insertion	(Springer, et al. 2016)	None	KX064685.1<br>Genome BLAST
	Cetartiodactyla	*D. leucas*	KC677009	E113G (exon 1) [a]	(Levenson and Dizon 2003)<br>(Meredith, et al. 2013)	None	Genome BLAST
	Cetartiodactyla	*E. robustus*	KC677001	Frameshift deletion	(Levenson and Dizon 2003)<br>(Meredith, et al. 2013)	None	Genome BLAST
	Cetartiodactyla	*E. glacialis*	AF545492	Frameshift deletion	(Levenson and Dizon 2003)	None	AF545492
	Cetartiodactyla	*E. japonica*	-	Frameshift deletion	(McGowen, et al. 2020)	None	Genome BLAST
	Cetartiodactyla	*G. melas*	AY228442	Frameshift insertion E113G (exon 1) [a]	(Newman and Robinson 2005)<br>(Peichl, et al. 2001)	Altered stop codon<br>TAA → CTG	XM_030853725.1
	Cetartiodactyla	*I. geoffrensis*	KC677011	E113G (exon 1) [a]	(Levenson and Dizon 2003)<br>(Meredith, et al. 2013)	Intron 1<br>AG → AC	Genome BLAST
	Cetartiodactyla	*K. breviceps*	KC677006	Frameshift deletion E113G (exon 1) [a]<br>Splice site mutation	(Levenson and Dizon 2003)<br>(Meredith, et al. 2013)	None	Genome BLAST

Cetartiodactyla	*L. obliquidens*	AB462245	E113G (exon 1) [a]	(Koito, et al. 2010; Springer, et al. 2016)	Altered stop codon<br>TAA → CTG	Genome BLAST
Cetartiodactyla	*L. vexillifer*	AUPI01142190	Frameshift insertion E113G (exon 1) [a]<br>Splice site mutation	(Springer, et al. 2016)<br>(Zhou, et al. 2013)	None	XM_007469685.1
Cetartiodactyla	*M. novaeangliae*	AY228440	Frameshift deletion E113G (exon 1) [a]	(Meredith, et al. 2013)<br>(Newman and Robinson 2005)	None	AY228440<br>Genome BLAST
Cetartiodactyla	*M. bidens*	KC677008	Frameshift deletion E113G (exon 1) [a]	(Levenson and Dizon 2003)<br>(Meredith, et al. 2013)	None	Genome BLAST
Cetartiodactyla	*M. monoceros*	KC677017	E113G (exon 1) [a]	(Meredith, et al. 2013)	None	XM_029234347.1
Cetartiodactyla	*N. asiaeorientalis*	-	-	-	E113G<br>(exon 1) [a]	Genome BLAST
Cetartiodactyla	*O. orca*	ANOL02032778	Frameshift deletion E113G (exon 1) [a]<br>Altered stop codon	(Emerling, et al. 2015)<br>(Levenson and Dizon 2003)<br>(Springer, et al. 2016)	None	Genome BLAST
Cetartiodactyla	*P. sinus*	AF545489	E113G (exon 1) [a]	(Levenson and Dizon 2003)	None	XM_032643523.1<br>Genome BLAST
Cetartiodactyla	*P. phocoena*	KC677010	E113G (exon 1) [a]	(Levenson and Dizon 2003)<br>(Meredith, et al. 2013)<br>(Peichl, et al. 2001)	None	Genome BLAST
Cetartiodactyla	*P. catodon*	KC677005	Frameshift insertion E113G (exon 1) [a]<br>Splice site mutation	(Levenson and Dizon 2003)<br>(Meredith, et al. 2013)<br>(Springer, et al. 2016)	None	XM_024132374.1
Cetartiodactyla	*P. minor*	KC677007	Frameshift deletion and insertion<br>E113G (exon 1) [a]	(Meredith, et al. 2013)	None	Genome BLAST
Cetartiodactyla	*P. blainvillei*	KC677012	Frameshift deletion and insertion<br>E113G (exon 1) [a]	(Levenson and Dizon 2003)<br>(Meredith, et al. 2013)	Intron 1<br>AG → AC	Genome BLAST
Cetartiodactyla	*S. chinensis*	AB462248	Frameshift deletion E113G (exon 1) [a]	(Koito, et al. 2010)	Altered stop codon<br>TAA → CTG	Genome BLAST
Cetartiodactyla	*T. aduncus*	JF504939	Frameshift deletion E113G (exon 1) [a]	(McGowen 2011)	Altered stop codon<br>TAA → CTG	Genome BLAST

Cetartiodactyla	*T. truncatus*	AF055458	Frameshift deletion E113G (exon 1) [a]<br>Altered stop codon	(Fasick, et al. 1998)<br>(Peichl, et al. 2001)<br>(Springer, et al. 2016)	None	XM_004311115.2
Cetartiodactyla	*Z. cavirostris*	KC677023	E113G (exon 1) [a]	(Meredith, et al. 2013)	None	Genome BLAST
Carnivora	*C. ursinus*	KT221559	Sequence intact [b]	(Emerling, et al. 2015)<br>(Peichl, et al. 2001)	None	Genome BLAST
Carnivora	*L. weddellii*	APMU01120147	Sequence intact [b]	(Emerling, et al. 2015)<br>(Levenson, et al. 2006)	None	XM_006745503.1
Carnivora	*N. schauinslandi*	-	Sequence intact [b]	(Levenson, et al. 2006)	None	XM_021699368.1<br>Genome BLAST
Carnivora	*O. rosmarus*	ANOP01012192	Sequence intact [b]	(Emerling, et al. 2015)<br>(Levenson, et al. 2006)	None	XM_004398023.2
Chiroptera	*D. rotundus*	MH664062	Frameshift deletion	(Simoes, et al. 2019)	Del 1bp 731	Genome BLAST
Chiroptera	*E. helvum*	AWHC01135359-60	Frameshift insertions and deletions<br>Premature stop codons	(Simoes, et al. 2019)<br>(Zhao, Rossiter, et al. 2009)	Intron 2<br>AG → CA	Genome BLAST
Chiroptera	*E. spelaea*	EU912375.1	Frameshift insertion<br>Splice site mutation	(Simoes, et al. 2019)<br>(Zhao, Rossiter, et al. 2009)	None	EU912375.1
Chiroptera	*H. armiger*	EU912368	Frameshift insertions and deletions<br>Premature stop codons	(Shen, et al. 2010)<br>(Simoes, et al. 2019)<br>(Zhao, Rossiter, et al. 2009)	Premature stop codon 1177-1179	Genome BLAST
Chiroptera	*M. lyra*	AWHB01305061-4<br>MH664073	Frameshift deletion	(Emerling, et al. 2015)<br>(Simoes, et al. 2019)	None	Genome BLAST
Chiroptera	*R. ferrumequinum*	EU912380	Frameshift deletions<br>Premature stop codons	(Simoes, et al. 2019)<br>(Zhao, Rossiter, et al. 2009)	None	Genome BLAST<br>EU912380
Chiroptera	*R. pusillus*	EU912382.1	Frameshift insertions and deletions<br>Premature stop codons	(Shen, et al. 2010)<br>(Simoes, et al. 2019)<br>(Zhao, Rossiter, et al. 2009)	None	EU912382.1
Chiroptera	*R. aegyptiacus*	MH664112	Frameshift deletions	(Simoes, et al. 2019)	None	XM_016148657.1<br>Genome BLAST
Chiroptera	*R. leschenaultii*	EU912381	Frameshift deletion	(Shen, et al. 2010)	None	EU912381.1

				(Simoes, et al. 2019)<br>(Zhao, Rossiter, et al. 2009)		
Pholidota	*M. javanica*	-	-	-	Del 19bp (657-675), 4bp (831-834), 20bp (842-861)<br>1bp insertion (700, 817, 890)<br>Altered stop codons<br>Premature stop codons (76-78, 544-546, 700-702)	XM_017652220.1
Pholidota	*M. pentadactyla*	JPTV01022983	Frameshift insertions and deletions<br>Premature stop codons<br>Altered stop codons	(Emerling and Springer 2015)	None	JPTV01022983<br>Genome BLAST
Eulipotyphla	*S. paradoxus* [c]	-	-	-	Del 1bp 103<br>Del 1bp 566<br>Del 2bp 780-781<br>Del 2bp 1020-1021	Genome BLAST
Erinaceomorpha	*E. europaeus*	AMDU01113654	Sequence intact [b]	(Emerling and Springer 2014)<br>(Glosmann, et al. 2001)	None	XM_007526304.1
Primates	*A. nancymaae*	DQ518238	Frameshift deletion<br>Premature stop codons	(Levenson, et al. 2007)	None	Genome BLAST
Primates	*C. medius*	DQ191908-12	Splice site mutation<br>Premature stop codon	(Tan, et al. 2005)<br>(Veilleux, et al. 2013)	None	Genome BLAST
Primates	*O. garnettii*	Ensembl73	Frameshift insertions and deletions<br>Premature stop codons	(Tan, et al. 2005)<br>(Wikler and Rakic 1990)	None	XM_012805124.1
Rodentia	*M. auratus*	AY029607-10	Frameshift deletion	(Calderone and Jacobs 1999)<br>(Von Schantz, et al. 1997)	None	XM_012805124.1<br>Genome BLAST
Rodentia	*M. pahari*		Sequence intact [b]	(Szél, et al. 1996)	None	XM_021189852.1
Rodentia	*N. ehrenbergi*	AY099455	Frameshift insertion and deletion	(David-Gray, et al. 2002)	None	AY099455.1

	Rodentia	*N. galili*	AXCX01145424	Frameshift insertion	(Fang, et al. 2014)	None	XM_008846382.1<br>Genome BLAST
M/LWS	Cingulata	*D. novemcinctus*	AAGV03294213<br>AAGV03294214	Frameshift deletions<br>Premature stop codons	(Emerling and Springer 2015)	None	Genome BLAST
	Afrosoricida	*C. asiatica*	AMDV01328078<br>KJ713966	Frameshift insertions and deletions<br>Premature stop codon<br>Splice site mutation	(Emerling and Springer 2014)	Altered start codon<br>ATG → GTG	Genome BLAST
	Cetartiodactyla	*B. mysticetus*	KC676796<br>KC676817<br>KC676839<br>KC676880<br>KC676900	Splice site mutation	(Meredith, et al. 2013)<br>(Springer, et al. 2016)	None	Genome BLAST
	Cetartiodactyla	*B. acutorostrata*	KC676797<br>KC676818<br>KC676840<br>KC676860<br>KC676881<br>KC676901	Frameshift deletions<br>Altered start codons	(Meredith, et al. 2013)<br>(Springer, et al. 2016)	None	XM_007185533.1<br>Genome BLAST
	Cetartiodactyla	*B. bonaerensis*	KX118310	Frameshift deletions<br>Altered start/stop codons	(Springer, et al. 2016)	None	Genome BLAST
	Cetartiodactyla	*E. robustus*	KC676824<br>KC676846<br>KC676866<br>KC676887<br>KC676907	Frameshift deletions	(Meredith, et al. 2013)	Altered start codons<br>ATG → GGG	Genome BLAST
	Cetartiodactyla	*E. japonica* [c]	-	-	-	Del 79bp 666-744<br>Del 7bp 994-1000<br>Intron 5 AG → GG	Genome BLAST
	Cetartiodactyla	*K. breviceps*	KC676806<br>KC676828<br>KC676850	Frameshift deletions<br>Splice site mutation	(Meredith, et al. 2013)	None	Genome BLAST

		KC676869<br>KC676911				
Cetartiodactyla	*M. novaeangliae*	KC676807<br>KC676829<br>KC676851<br>KC676870<br>KC676891<br>KC676912	Frameshift deletions	(Meredith, et al. 2013)	Altered start codons<br>ATG → GGG<br>Intron 5<br>GT → AT	Genome BLAST
Cetartiodactyla	*M. bidens*	KC676808<br>KC676830<br>KC676852<br>KC676871<br>KC676892<br>KC676913	Frameshift insertion	(Meredith, et al. 2013)	None	Genome BLAST
Cetartiodactyla	*P. catodon*	KC676812<br>KC676834<br>KC676855<br>KC676875<br>KC676895<br>KC676916	Frameshift deletions<br>Altered stop codons	(Meredith, et al. 2013)<br>(Springer, et al. 2016)	None	XM_007113595.1<br>Genome BLAST
Rodentia	*E. lutescens* [c]	-	-	-	Premature stop codon 679-681<br>Intron 2: GT → AT	Genome BLAST

a. E113G replacement (bovine rhodopsin numbering), which could impair normal pigment function (Levenson and Dizon 2003; Meredith, et al. 2013).
b. Complete coding sequence, however, considered potential pseudogene based on other evidence.
c. Inactivating mutations found in this study verified by raw reads in supplementary table S2.

Supplementary table S4. Parameters estimated by CODEML for branches with ω value greater than one

Gene	Branch number *	ω	Nonsynonymous substitutions	Synonymous substitutions
SWS1	1	∞	15.7	0.0
	2	∞	1.0	0.0
	3	∞	1.0	0.0
	4	1.08	20.0	6.0
	5	∞	1.0	0.0
	6	∞	1.0	0.0
	7	∞	5.0	0.0
	8	1.62	1.0	0.2
	9	∞	3.0	0.0
	10	∞	2.3	0.0
	11	∞	3.9	0.0
	12	∞	1.0	0.0
	13	∞	1.0	0.0
	14	∞	3.1	0.0
	15	1.77	11.4	2.1
	16	∞	1.0	0.0
	17	1.29	4.1	1.0
	18	∞	2.1	0.0
M/LWS	1	∞	1.0	0.0
	2	∞	1.2	0.0
	3	∞	1.0	0.0
	4	∞	1.0	0.0
	5	∞	1.0	0.0
	6	∞	3.1	0.0
RH1	1	3.34	6.3	0.3
	2	1.47	9.8	1.2
	3	∞	4.0	0.0
	4	∞	1.0	0.0
	5	∞	2.0	0.0
	6	∞	1.1	0.0
	7	∞	0.9	0.0
	8	∞	3.0	0.0
	9	∞	1.0	0.0
	10	∞	1.0	0.0
	11	∞	1.7	0.0
	12	∞	1.0	0.0
	13	∞	4.7	0.0
	14	∞	1.8	0.0

* Numbering of the branches is shown in fig. 1.

Supplementary table S5. Site-directed mutagenesis primers for *SWS1* and *RH1* genes

**Name**	**Sequence (5′ → 3′)**
Mus-*SWS1*-86A-F	CTCGGGGGCTTCCTCTGCATCTTCTCTGTC
Mus-*SWS1*-86A-R	GACAGAGAAGATGCA GGCGAGGAAGCCCCCGAG
Mus-*SWS1*-93N-F	ATCTTCTCTGTCTTCAATGTCTTCATCGCCAGC
Mus-*SWS1*-93N-R	GCTGGCGATGAAGACATTGAAGACAGAGAAGAT
Mus-*SWS1*-86A93N-F	GGGCTTCCTCGCCTGCATCTTCTCTGTCTTCAATGTCTTCATCG
Mus-*SWS1*-86A93N-R	CGATGAAGACATTGAAGACAGAGAAGATGCAGGCGAGGAAGCCC
Pika-*SWS1*-86F-F	CTGGCCGGCTTCCTGTTCTGCATCTTCAGCGTGTT
Pika-*SWS1*-86F-R	AACACGCTGAAGATGCAGAACAGGAAGCCGGCCAG
Pika-*SWS1*-93T-F	TGCATCTTCAGCGTGTTCACAGTGTTCCTGAGCAGCTG
Pika-*SWS1*-93T-R	CAGCTGCTCAGGAACACTGTGAACACGCTGAAGATGCA
Pika-*SWS1*-86F93T-F	CGGCTTCCTGTTCTGCATCTTCAGCGTGTTCACAGTGTTCCTGAG
Pika-*SWS1*-86F93T-R	CTCAGGAACACTGTGAACACGCTGAAGATGCAGAACAGGAAGCCG
Bov-*RH1*-195S-F	CGATTACTACACCCCCTCGGAGGAGACCAATAATG
Bov-*RH1*-195S-R	CATTATTGGTCTCCTCCGAGGGGGTGTAGTAATCG
Bov-*RH1*-195T-F	CGATTACTACACCCCCACCGAGGAGACCAATAATG
Bov-*RH1*-195T-R	CATTATTGGTCTCCTCGGTGGGGGTGTAGTAATCG
Physeter-*RH1*-195H-F	CGACTACTACACCCTGCACCCCGAGGTGAACAACG
Physeter-*RH1*-195H-R	CGTTGTTCACCTCGGGGTGCAGGGTGTAGTAGTCG
Physeter-*RH1*-195K-F	CGACTACTACACCCTGAAGCCCGAGGTGAACAACG
Physeter-*RH1*-195K-R	CGTTGTTCACCTCGGGCTTCAGGGTGTAGTAGTCG

References

Ahnelt PK, Schubert C, Kübber-Heiss A, Schiviz A, Anger E. 2006. Independent variation of retinal S and M cone photoreceptor topographies: a survey of four families of mammals. Vis Neurosci 23:429-435.

Arbogast P, Glösmann M, Peichl L. 2013. Retinal cone photoreceptors of the deer mouse Peromyscus maniculatus: development, topography, opsin expression and spectral tuning. PLoS One 8:e80910.

Bennie JJ, Duffy JP, Inger R, Gaston KJ. 2014. Biogeography of time partitioning in mammals. Proc Natl Acad Sci U S A 111:13727-13732.

Borges R, Johnson WE, O'Brien SJ, Gomes C, Heesy CP, Antunes A. 2018. Adaptive genomic evolution of opsins reveals that early mammals flourished in nocturnal environments. BMC Genomics 19:121.

Calderone JB, Jacobs GH. 1999. Cone receptor variations and their functional consequences in two species of hamster. Vis Neurosci 16:53-63.

Carroll J, Murphy CJ, Neitz M, Ver Hoeve JN, Neitz J. 2001. Photopigment basis for dichromatic color vision in the horse. J Vis 1:2-2.

Carvalho LS, Cowing JA, Wilkie SE, Bowmaker JK, Hunt DM. 2006. Shortwave visual sensitivity in tree and flying squirrels reflects changes in lifestyle. Curr Biol 16:R81-R83.

Carvalho LS, Davies WL, Robinson PR, Hunt DM. 2012. Spectral tuning and evolution of primate short-wavelength-sensitive visual pigments. Proc R Soc B 279:387-393.

David - Gray ZK, Bellingham J, Munoz M, Avivi A, Nevo E, Foster RG. 2002. Adaptive loss of ultraviolet - sensitive/violet - sensitive (UVS/VS) cone opsin in the blind mole rat (*Spalax ehrenbergi*). Eur J Neurosci 16:1186-1194.

Deeb SS, Wakefield MJ, Tada T, Marotte L, Yokoyama S, Marshall Graves JA. 2003. The cone visual pigments of an Australian marsupial, the tammar wallaby (*Macropus eugenii*): sequence, spectral tuning, and evolution. Mol Biol Evol 20:1642-1649.

Dungan SZ, Kosyakov A, Chang BS. 2016. Spectral tuning of killer whale (*Orcinus orca*) rhodopsin: evidence for positive selection and functional adaptation in a cetacean visual pigment. Mol Biol Evol 33:323-336.

Emerling CA, Huynh HT, Nguyen MA, Meredith RW, Springer MS. 2015. Spectral shifts of mammalian ultraviolet-sensitive pigments (short wavelength-sensitive opsin 1) are associated with eye length and photic niche evolution. Proc R Soc B 282:20151817.

Emerling CA, Springer MS. 2014. Eyes underground: regression of visual protein networks in subterranean mammals. Mol Phylogenet Evol 78:260-270.

Emerling CA, Springer MS. 2015. Genomic evidence for rod monochromacy in sloths and armadillos suggests early subterranean history for Xenarthra. Proc R Soc B 282:20142192.

Fang X, Nevo E, Han L, Levanon EY, Zhao J, Avivi A, Larkin D, Jiang X, Feranchuk S, Zhu Y. 2014. Genome-wide adaptive complexes to underground stresses in blind mole rats Spalax. Nat Commun 5:1-11.

Fasick JI, Applebury ML, Oprian DD. 2002. Spectral tuning in the mammalian short-wavelength sensitive cone pigments. Biochemistry 41:6860-6865.

Fasick JI, Cronin TW, Hunt DM, Robinson PR. 1998. The visual pigments of the bottlenose dolphin (*Tursiops truncatus*). Vis Neurosci 15:643-651.

Glosmann M, Harlfinger PJ, Ahnelt PK. 2001. S opsin-like immunoreactivity is localized to bipolar cells but not cone photoreceptors in the European hedgehog. Invest Ophthalmol Vis Sci 42:S362.

Gutierrez EA, Castiglione GM, Morrow JM, Schott RK, Loureiro LO, Lim BK, Chang BS. 2018. Functional shifts in bat dim-light visual pigment are associated with differing echolocation abilities and reveal molecular adaptation to photic-limited environments. Mol Biol Evol 35:2422-2434.

Gutierrez EA, Schott RK, Preston MW, Loureiro LO, Lim BK, Chang BS. 2018. The role of ecological factors in shaping bat cone opsin evolution. Proc R Soc B 285:20172835.

Herbin M, Reperant J, Cooper HM. 1994. Visual system of the fossorial mole-lemmings, *Ellobius talpinus* and *Ellobius lutescens*. J Comp Neurol 346:253-275.

Hunt DM, Chan J, Carvalho LS, Hokoc JN, Ferguson MC, Arrese CA, Beazley LD. 2009. Cone visual pigments in two species of South American marsupials. Gene 433:50-55.

Ishengoma E, Agaba M, Cavener DR. 2017. Evolutionary analysis of vision genes identifies potential drivers of visual differences between giraffe and okapi. PeerJ 5:e3145.

Jacobs GH, Deegan JF, Crognale MA, Fenwick JA. 1993. Photopigments of dogs and foxes and their implications for canid vision. Vis Neurosci 10:173-180.

Koito T, Kubokawa K, Tanabe S, Miyazaki N. 2010. Phylogenetic analyses in cetacean species of the family Delphinidae using a short wavelength sensitive opsin gene sequence. Fish Sci 76:571-576.

Levenson DH, Dizon A. 2003. Genetic evidence for the ancestral loss of short-wavelength-sensitive cone pigments in mysticete and odontocete cetaceans. Proc Biol Sci 270:673-679.

Levenson DH, Fernandez-duque E, Evans S, Jacobs GH. 2007. Mutational changes in S-cone opsin genes common to both nocturnal and cathemeral Aotus monkeys. Am J Primatol 69:757-765.

Levenson DH, Ponganis PJ, Crognale MA, Deegan JF, Dizon A, Jacobs GH. 2006. Visual pigments of marine carnivores: pinnipeds, polar bear, and sea otter. J Comp Physiol A 192:833-843.

Liu Y, Chi H, Li L, Rossiter SJ, Zhang S. 2018. Molecular data support an early shift to an intermediate-light niche in the evolution of mammals. Mol Biol Evol 35:1130-1134.

Liu Y, Cui Y, Chi H, Xia Y, Liu H, Rossiter SJ, Zhang S. 2019. Scotopic rod vision in tetrapods arose from multiple early adaptive shifts in the rate of retinal release. Proc Natl Acad Sci U S A 116:12627-12628.

McGowen MR. 2011. Toward the resolution of an explosive radiation—a multilocus phylogeny of oceanic dolphins (Delphinidae). Mol Phylogenet Evol 60:345-357.

McGowen MR, Tsagkogeorga G, Williamson J, Morin PA, Rossiter, J. S. 2020. Positive selection and inactivation in the vision and hearing genes of cetaceans. Mol Biol Evol 37:2069-2083.

Meredith RW, Gatesy J, Emerling CA, York VM, Springer MS. 2013. Rod monochromacy and the coevolution of cetacean retinal opsins. PLoS Genet 9:e1003432.

Moritz GL, Lim NT, Neitz M, Peichl L, Dominy NJ. 2013. Expression and evolution of short wavelength sensitive opsins in colugos: a nocturnal lineage that informs debate on primate origins. Evol Biol 40:542-553.

Newman LA, Robinson PR. 2005. Cone visual pigments of aquatic mammals. Vis Neurosci 22:873-879.

Onishi A, Koike S, Ida-Hosonuma M, Imai H, Shichida Y, Takenaka O, Hanazawa A, Komatsu H, Mikami A, Goto S. 2002. Variations in long-and middle-wavelength-sensitive opsin gene loci in crab-eating monkeys. Vision Res 42:281-292.

Parry JW, Poopalasundaram S, Bowmaker JK, Hunt DM. 2004. A novel amino acid substitution is responsible for spectral tuning in a rodent violet-sensitive visual pigment. Biochemistry 43:8014-8020.

Peichl L, Behrmann G, Kröger RHH. 2001. For whales and seals the ocean is not blue: a visual pigment loss in marine mammals. Eur J Neurosci 13:1520-1528.

Sadier A, Davies KT, Yohe LR, Yun K, Donat P, Hedrick BP, Dumont ER, Davalos LM, Rossiter SJ, Sears KE. 2018. Multifactorial processes underlie parallel opsin loss in neotropical bats. eLife 7:e37412.

Shen Y, Liu J, Irwin D, Zhang Y. 2010. Parallel and convergent evolution of the dim-light vision gene RH1 in bats (Order: Chiroptera). PLoS One 5:e8838.

Shimmin LC, Miller J, Tran HN, Li W. 1998. Contrasting levels of DNA polymorphism at the autosomal and X-linked visual color pigment loci in humans and squirrel monkeys. Mol Biol Evol 15:449-455.

Simoes BF, Foley NM, Hughes GM, Zhao H, Zhang S, Rossiter SJ, Teeling EC. 2019. As blind as a bat? Opsin phylogenetics illuminates the evolution of color vision in bats. Mol Biol Evol 36:54-68.

Southall KD, Oliver GW, Lewis JW, Le Boeuf BJ, Levenson DH. 2002. Visual pigment sensitivity in three deep diving marine mammals. Mar Mamm Sci 18:275-281.

Springer MS, Emerling CA, Fugate N, Patel R, Starrett J, Morin PA, Hayashi C, Gatesy J. 2016. Inactivation of cone-specific phototransduction genes in rod monochromatic cetaceans. Front. Ecol. Evol 4:61.

Szél Á, Röhlich P, Caffé AR, Van Veen T. 1996. Distribution of cone photoreceptors in the mammalian retina. Microsc Res Tech 35:445-462.

Tan Y, Yoder AD, Yamashita N, Li W. 2005. Evidence from opsin genes rejects nocturnality in ancestral primates. Proc Natl Acad Sci U S A 102:14712-14716.

Veilleux CC, Louis Jr EE, Bolnick DA. 2013. Nocturnal light environments influence color vision and signatures of selection on the *OPN1SW* opsin gene in nocturnal lemurs. Mol Biol Evol 30:1420-1437.

Von Schantz M, Argamaso-Hernan SM, Szél Á, Foster RG. 1997. Photopigments and photoentrainment in the Syrian golden hamster. Brain Res 770:131-138.

Wikler KC, Rakic P. 1990. Distribution of photoreceptor subtypes in the retina of diurnal and nocturnal primates. J Neurosci 10:3390-3401.

Wu Y, Wang H, Hadly EA. 2017. Invasion of ancestral mammals into dim-light environments inferred from adaptive evolution of the phototransduction genes. Sci Rep 7:1-9.

Yokoyama S, Radlwimmer FB, Kawamura S. 1998. Regeneration of ultraviolet pigments of vertebrates. FEBS Lett 423:155-158.

Zhao H, Rossiter SJ, Teeling EC, Li C, Cotton JA, Zhang S. 2009. The evolution of color vision in nocturnal mammals. Proc Natl Acad Sci U S A 106:8980-8985.

Zhao H, Ru B, Teeling EC, Faulkes CG, Zhang S, Rossiter SJ. 2009. Rhodopsin molecular evolution in

mammals inhabiting low light environments. PLoS One 4:e8326.
Zhou X, Sun F, Xu S, Fan G, Zhu K, Liu X, Chen Y, Shi C, Yang Y, Huang Z. 2013. Baiji genomes reveal low genetic variability and new insights into secondary aquatic adaptations. Nat Commun 4:1-6.
